# Effectiveness of smoking cessation on the high-risk population of lung cancer with early screening: a systematic review and meta-analysis of randomized controlled trials until January 2022

**DOI:** 10.1186/s13690-023-01111-5

**Published:** 2023-06-03

**Authors:** Simin Huang, Oufeng Tang, Xutong Zheng, Hui Li, Yuxin Wu, Liu Yang

**Affiliations:** 1 School of Nursing, Fujian University of Traditional Chinese Medicine-Fuzhou, Fuzhou, China; 2 Department of Anesthesiology, West China Hospital of Sichuan University/West China School of Nursing, Sichuan University-Chengdu, Chengdu, China; 3 Ji’an College-Ji’an, Ji’an, China

**Keywords:** Early detection of cancer, Lung neoplasms, Smoking cessation, Systematic review, Meta-analysis, Randomized controlled trial

## Abstract

**Background:**

Lung cancer has always been the malignant tumor with the highest incidence rate. Smoking is the most important risk factor for lung cancer. Although potential positive effects of smoking cessation interventions on the high-risk population of lung cancer have been observed, evidence of its definitive effect remains uncertain. This study aimed to summarize the evidence related to the effects and safety of smoking cessation interventions for the high-risk population of lung cancer.

**Methods:**

A systematic literature search was conducted through the following seven databases: PubMed, Embase, Web of Science, CENTRAL, CINAHL, PsycINFO, and Science Direct. Screening and assessment for risk of bias were conducted by two independent reviewers. Meta-analysis was performed for the 7-day-point prevalence of smoking abstinence and continuous smoking abstinence using RevMan 5.3 software.

**Results:**

Meta-analysis results show that in the 7-day-point prevalence of smoking abstinence (by patient-reported outcome): individualized intervention was significantly higher than that of the standard care [RR = 1.46, 95%CI = (1.04,2.06), *P* < 0.05]. Moreover, the smoking cessation interventions were significantly elevated than that of standard care [RR = 1.58, 95%CI = (1.12, 2.23), *P* < 0.05] within 1–6 month follow-up time. In line with the findings in cigarette smoking, the continuous smoking abstinence of E-cigarettes (biochemical verified): E-cigarettes were significantly higher than that of the standard care [RR = 1.51, 95%CI = (1.03, 2.21), *P* < 0.05], and within 1–6 month follow-up time, the smoking cessation interventions were significantly greater than that of standard care [RR = 1.51, 95%CI = (1.03, 2.21), *P* < 0.05]. Publication bias was detected possibly.

**Conclusions:**

The results of this systematic review show that smoking cessation intervention is effective for long-term lung cancer high-risk smokers who participate in early screening, of which E-cigarettes are the best, followed by individual smoking cessation.

**Trial registration:**

A review protocol was developed and registered in the International Prospective Register of Systematic Reviews (PROSPERO). Trial registration: CRD42019147151. Registered 23 June 2022.

**Supplementary Information:**

The online version contains supplementary material available at 10.1186/s13690-023-01111-5.

## Background

Lung cancer has been the most common malignant tumor, according to the most recent statistics on cancer in the world provided by the American Cancer Society. It is also the leading cause of death among all cancer patients, accounting for 18.4% of all cancer deaths globally with an 18.6% average 5-year survival rate [1, 2]. The family, economic, and social burdens caused by lung cancer are increasing worldwide [3, 4]. The leading incidence and mortality of lung cancer are largely attributed to smoking behavior. According to the Global Burden of Disease Study, the number of smokers increased to 1.1 billion in 2019 worldwide [5]. The International Agency for Research on Cancer showed that over 4000 chemical agents in cigarette smoke, 60 of which are well-known carcinogens [6], long-term smoking increases the risk of lung cancer by 10–30 folds compared to non-smokers, which makes smoking one of the most important risk factors for lung cancer [7, 8]. In addition, cigarette smoke may have the ability not only to promote lung cancer but also influence directly or indirectly the efficacy and the tolerability of many chemotherapeutics through complex pharmacokinetic [9]. It is known that smoking cessation can reduce the risk of lung cancer. Ex-smokers who have quit smoking for 15 years showed 38% lower mortality when they developed lung cancer compared to current smokers [10], indicating that smoking cessation has a major beneficial effect on the survival of lung cancer, smoking cessation program has to be considered crucial along with specific therapy for cancer [9]. The U.S. Centers for Medicare & Medicaid Services and The Tobacco Use Treatment Association strongly recommend smoking cessation intervention as a precondition for initiating early screening programs for lung cancer [11, 12]. One of the key causes of lung cancer’s poor prognosis is the challenge of early diagnosis. Approximately 70% of lung cancer patients were diagnosed at an advanced stage, and around one-third of patients died within 3 months after diagnosis [13]. It is estimated that the five-year survival rate of lung cancer can be increased by roughly 75% with an early diagnosis which may subsequently reduce lung cancer mortality [14]. The National Lung Screening Trial reported that early lung cancer screening with low-dose computed tomography reduced the mortality of patients with lung cancer by 20% [15]. The National Comprehensive Cancer Network Guidelines also pointed out that conducting early screening and diagnosis for those who are at high risk of developing lung cancer is crucial, especially for people aged 55 to 74 years with a minimum of 30 pack years of smoking [16]. Early screening provides the option to detect lung cancer earlier before the disease develops into an advanced stage. In the meantime, the implementation of early screening aids in drawing long-term smokers’ attention to the critical role smoking plays in an elevated risk of lung cancer. This could motivate smokers who are at high risk of developing lung cancer to give up and make themselves open to smoking cessation treatments [17–19]. However, studies have shown that most smokers who participate in lung cancer screening continue to smoke [20]. Therefore, health care workers have been actively looking for a better system for carrying out smoking cessation intervention, encouraging the high-risk population for lung cancer to quit smoking effectively and reduce the incidence of lung cancer.

Although existing systematic reviews found that smoking cessation interventions included long-term use of pharmacotherapy, high tech ereferral systems, national quitting programmes, quitting apps, enhanced counselling, and an opt-out referral system, etc. [21] and smoking cessation intervention has a potentially positive effect on the prognosis of long-term high-risk smokers with lung cancer, a comprehensive search and adequate reporting of clinical randomized controlled trials are lacking [19, 22, 23]. There are very few reviews have provided quantitative analysis of existing data to demonstrate the effect of smoking cessation intervention on lung cancer in long-term high-risk smokers**.** Systematic reviews or meta-analysis including high-quality randomized controlled trials is considered to be a valuable addition to the field. Therefore, the purpose of this systematic review and meta-analysis is to analyze randomized controlled trials of smoking cessation intervention in long-term high-risk smokers who participated in early screening to determine which smoking cessation methods are more effective in reducing lung cancer incidence in terms of quantity and quality.

## Methods

This study was conducted under the standards of The Preferred Reporting Items for Systematic Reviews and Meta-Analyses (PRISMA) statement [24] and registered in the International Prospective Register of Systematic Review database (Trial registration: CRD42019147151. Registered 23 June 2022).

### Data sources and search strategy 

Two authors (Huang and Tang) performed the search independently. A total of 7 electronic databases, including PubMed, Excerpta Medica Database (Embase), Web of Science, Cochrane Library (Cochrane Central Register of Controlled Trials, CENTRAL), Cumulative Index to Nursing and Allied Health Literature (CINAHL), PsycINFO, and Science Direct, were searched for relevant studies published before January 2022. No language restrictions were applied. We also read the reference of the included studies to find potential eligible references. The search used a combination of MESH terms, keywords, and free words, such as ‘Tobacco Use Cessation’, ‘Smoking Cessation’, ‘Tobacco Use Disorder’, ‘Early Detection of Cancer’, ‘Lung Neoplasms’, and ‘Randomized Controlled Trial’. The detailed search strategy used in this study can be found in the Additional file 1.

### Eligibility criteria

Studies were considered eligible if they met all the following criteria: (a) design in the randomized controlled trial; (b) participants: smokers who smoked ≥ 30 packs/year and stopped smoking < 15 years, regardless of sex; (c) interventions: studies comparing smoking cessation interventions to standard care, including authoritative smoking cessation materials, placebo or other therapies with similar co-interventions between intervention and control groups; types of smoking cessation interventions can be any network resource, individualized intervention, and replacement therapy; (d) outcome measures: the main outcome measures were smoking abstinence, which can be verified by patient-reported or biochemical verification. The outcome measures included in the analyzed studies were a 7-day-point prevalence of smoking abstinence (smoking cessation rate within 7 days before follow-up) and continuous smoking abstinence (never smoking during the period from the date of quitting smoking till the end of follow-up); the secondary outcome measure was side effects. The study was excluded when met the following criteria: (a) case reports, case–control studies, reviews, protocols, or animal experimental studies; (b) studies without smoking abstinence as part of the outcome measure; (c) duplicated publications; and (d) incomplete or unavailable data.

### Study selection

All studies were independently screened by two co-authors (Huang and Tang) according to the inclusion and exclusion criteria. Two co-authors (Huang and Tang) used the homemade standardized table for data extraction and cross-checking. If any discrepancies were generated, a third person (Yang) was involved to make the decision. The original authors were approached for the confirmation of unclear details for some studies when it was necessary.

### Data extraction

The data from each study were extracted using a standardized table containing the following information: (a) first author, publication date, and study sites; (b) demographic data of subjects, such as type of sample, age, and sex of subjects; (c) intervention program, such as smoking cessation interventions, duration of treatment, frequency of treatment and follow-up; (d) outcome measures; and (e) smoking cessation interventions-related adverse events.

### Quality assessment 

The risk of bias was assessed for each included study according to The Cochrane Handbook for Systematic Reviews of Interventions Version 6.1 [25] which includes the evaluation in the following seven aspects: random sequence generation, allocation concealment, blind method of participants, blind method of result evaluation, incomplete result data, selective result report, and other deviations [25]. The individual studies were rated as low, unclear, or high bias risk for the mentioned 7 aspects. The risk of bias assessment was independently completed by two reviewers (Huang and Tang) and cross-checked. When there was disagreement regarding the study’s bias assessment, Yang was brought in as a third party to resolve the issue.

### Data synthesis and presentation 

RevMan 5.3 (developed by the Cochrane Collaboration) was used for data analysis. Heterogeneity was assessed using Q statistics and the *I*^2^ index. When the heterogeneity test resulted in *P* ≥ 0.1 with *I*^2^ < 50%, the fixed-effects model was used for meta-analysis. When the heterogeneity test resulted in *P* < 0.1 with *I*^2^ ≥ 50%, a subgroup analysis or sensitivity analysis was performed to determine potential sources of clinical heterogeneity before the meta-analysis was conducted with a random-effects model [25]. Descriptive analysis was performed on studies with significant clinical heterogeneity when *I*^2^ ≥ 75%. Relative risk (RR) was used for dichotomous data. For continuous variables, the mean difference (MD) was calculated when outcome measures were collected using the same research instrument [25]. Otherwise, the standardized mean difference (SMD) was utilized. *P* < 0.05 was considered statistically significant.

## Results

**Fig. 1:**
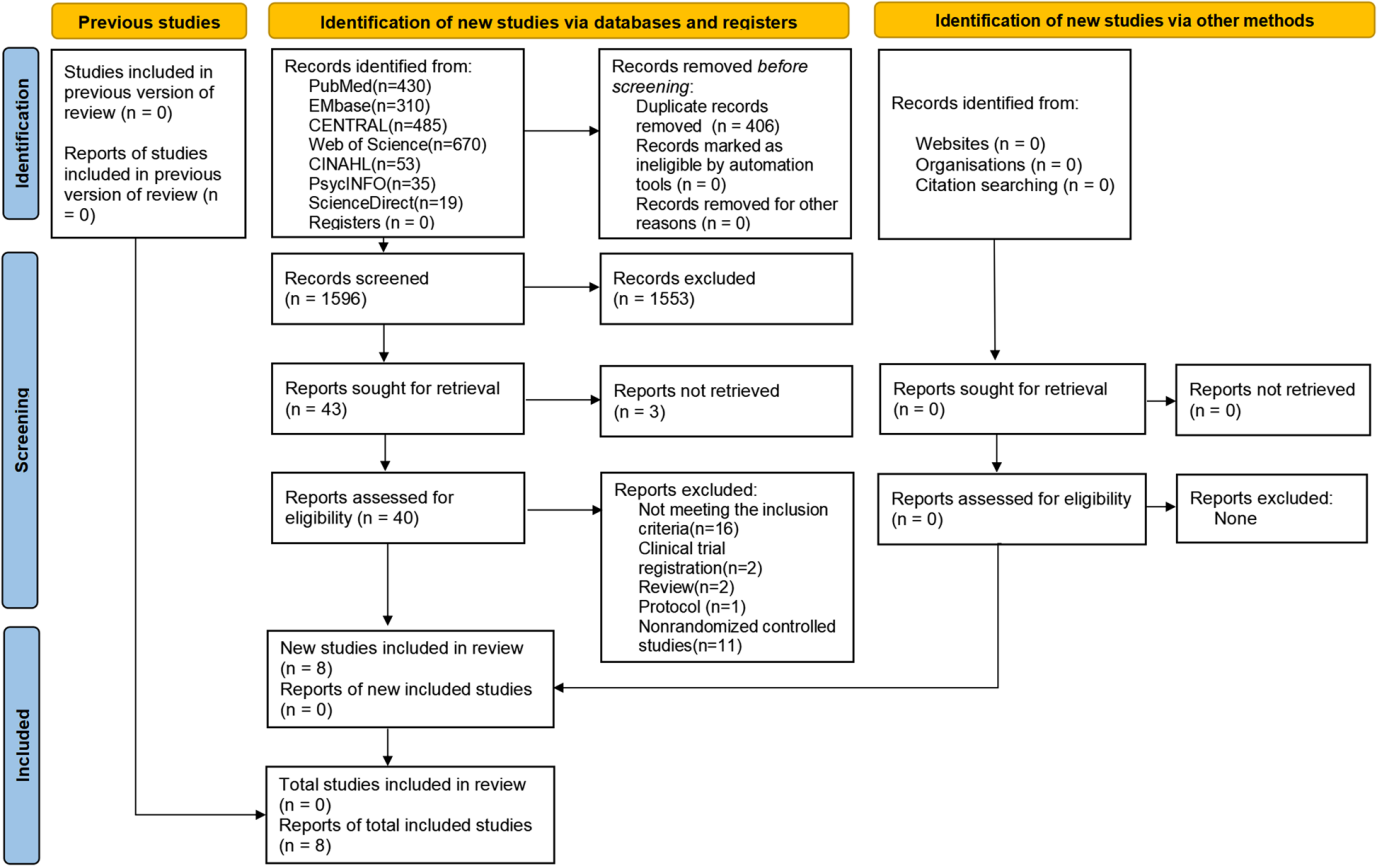
PRISMA flow diagram, the effectiveness of smoking cessation on the high-risk population of lung cancer with early screening: a systematic review and meta analysis until January, 2022

A total of 2002 studies were found from the initial search, from which 1596 unique potentially eligible studies were selected after removing the duplicate. After reading the title and abstract of those studies, 1553 studies were excluded for inconformity to the inclusion and exclusion criteria. The next step was to thoroughly read the full text of 43 records to determine their eligibility. Of these, 35 studies were found to be ineligible because they were clinical trial registrations (*n* = 2), reviews (*n* = 2), methodology publications (*n* = 1), or non-randomized controlled studies (*n* = 11). Additionally, 16 records were found to not meet the inclusion criteria. Finally, 8 studies were identified for the subsequent meta-analysis. The selected PRISMA flow chart is shown in Fig. 1.

### Summary of study details and participant characteristics

As a result, 8 studies were included in this study for the following analysis, all of which were English studies published between 2004 and 2022 [26–33]. A total of 988 participants aged 57 to 63 were included. Two were from the United States [31, 33], two were from Italy [28, 30], two were from Canada [27, 29], one was from Australia [32] and the last one was from England [26]. Among them, Tremblay et. al. published a follow-up article [27] of their work published in 2019 [29] with an extra set of data collected at a time point that was later than in their previous publication. Similar to the articles from Masiero et. al [30]. and Lucchiari et al. [28] which were the same study with 3 and 6 months-observation time individually. Therefore, these four articles were considered as two studies, “S4 Tremblay, et al.” [27, 29] and “Masiero & Lucchiari, et al.” [28, 30] in this analysis. All studies clearly described the specific intervention methods of smoking cessation interventions. The study of Clark M.M. et. al [33] came from network resources vs standard care. Four studies [26, 27, 29, 31, 32] were selected from individualized intervention vs standard care, which provided personalized smoking cessation services for smokers by taking advantage of inquiring about their smoking motivation and addiction degree, combining the screening results, including counseling, suggestions, and interviews. The S5 Masiero & Lucchiari study [28, 30] used E-cigarette + low-intensity telephone consultation and placebo + low-intensity telephone consultation as smoking cessation interventions to provide smoking cessation intervention. The comparison between “E-cigarette + low-intensity telephone consultation” and “low-intensity telephone consultation” was defined as the group (A), and the comparison between “E-cigarette + low-intensity telephone consultation” versus “placebo + low intensity telephone consultation” was defined as the group (B). Taylor KL et. al [31] reported that the time of intervention lasted for 15–20 min, the duration of intervention was one day which lasted for 6 weeks. The S5 Masiero & Lucchiari study [28, 30] reported that the duration of intervention lasted 12 weeks. The main outcome indicators included in the literature were 7-day-point prevalence and continuous smoking abstinence, which were evaluated by the patient-reported outcome and biochemical verification. Concerning the 7-day-point prevalence, four studies [26, 27, 29, 32, 33] used patient-reported outcomes, and other studies [31] used both patient-reported outcomes and biochemical verification. Regarding continuous smoking abstinence, Clark M.M. [33] used patient-reported outcome, S5 Masiero & Lucchiari study used biochemical verification [28, 30] and the Tremblay study used both patient-reported outcome and biochemical verification. In addition, Masiero & Lucchiari [28, 30] reported smoking cessation interventions-related adverse events, including burning throat, cough, nausea, headache, insomnia, stomachache, confusion, and dyspnea. The 7-day-point prevalence was recorded by patient-reported with the follow-up time of 1–6 months for 4 studies [26, 27, 29, 31, 33] respectively, within 6–12 months for two studies [27, 29, 32] and within 12–24 months for one study [27, 29]. Of note, the “Tremblay, et al.” study used 3 different time points of follow-up data collection, the follow-up time within 1–6 months, 6-12 months, and 12–24 months are defined as S4 Tremblay, et al. (a), S4 Tremblay, et al. (b) and S4 Tremblay, et al. (c) respectively. In the continuous smoking abstinence, by the patient-reported outcome, Tremblay study [27, 29] was with follow-up time within 1–6 months, 6–12 months, and 12–24 months, respectively; one study [28, 30] with a follow-up time of 1–6 months was verified by biochemical verification. In S5 Masiero & Lucchiari [28, 30] the data of two follow-up periods were collected by biochemical verification. In this study, “S5 Masiero & Lucchiari, et al. (A)” for three months was defined as “S5 Masiero & Lucchiari, et al. (a)”, “S5 Masiero & Lucchiari, et al. (B)” for three months was defined as “S5 Masiero & Lucchiari, et al. (b)”, “S5 Masiero & Lucchiari, et al. (A)” for 6 months was defined as “S5 Masiero & Lucchiari, et al. (c)”, and “S5 Masiero & Lucchiari, et al. (B)” for 6 months was defined as “S5 Masiero & Lucchiari, et al. (d)”. All studies include a report for follow-up after the intervention. The details are shown in Table 1.

**Table 1:** The characteristics of the included trials in the effectiveness of smoking cessation on the high-risk population of lung cancer with early screening: a systematic review and meta analysis until January, 2022

**Study and setting**	**Sample (T/C)**	**Age (yr) (T/C)**	**Gender M/F (T/C)**	**Fagestrom test**		**pack-years**		**eCO (ppm value)**	
				**T**	**C**	**T**	**C**	**T**	**C**
Buttery et al., 2022, England [26]	65/50	62.58 ± 6.09/ 61.68 ± 5.53	T:35/30 C:24/26	NR	NR	NR	NR	NR	NR
Tremblay et al., 2019 & 2021, Canada [27, 29]	171/174	62.0 ± 5.6/ 61.9 ± 5.2	T:73/98 C:86/88	5.0(4.0–7.0)^d^	5.5(4.0–7.0)^d^	42.3(14.1)^c^	44.0(15.1)^c^	NR	NR
Masiero & Lucchiari et al., 2020, Italy [28, 30]	T1 = 70/ T2 = 70/ C = 70	62.8 ± 4.587	140/70	T1:4.5(1.788)^c^ T2:4.4(1.878)^c^	4.1(1.954)^c^	NR	NR	T1: 15.2(5.275)^c^ T2:14.6(5.942)^c^	14.6(6.993)^c^
Taylor et al., 2017, America [31]	46/46	60.4 ± 5.1/ 60.1 ± 5.7	T:21/25 C:19/27	4.1(1.9)^c^	4.6(2.0)^c^	43.8(23.7)^c^	50.3(20.4)^c^	NR	NR
Marshall et al., 2016, Australia [32]	28/27	63.0 ± 6.0/ 63.0 ± 5.0	T:18/10 C:17/10	6.0(4)^d^	6.0(3)^d^	57.5(28)^d^	57.5(32)^d^	NR	NR
Clark et al., 2004, America [33]	85/86	57.8 ± 5.2/ 57.0 ± 5.3	T:46/39 C:41/45	56/85^a^ 29/85^b^	51/86^a^ 35/86^b^	NR	NR	NR	NR
**Study and setting**	**Intervention**			**Degree of intervention**	**Duration of intervention**		**Outcome/Scale**	**Adverse events**	**Follow-up time (M)**
	**T**	**C**							
Buttery et al., 2022, England [26]	individualized intervention	SC		NR	NR		7-day-point prevalence/PRO	NR	3
Tremblay et al., 2019 & 2021, Canada [27, 29]	individualized intervention	SC		NR	NR		7-day-point prevalence/PRO continuous smoking abstinence/PRO, BV	NAE	24
Masiero & Lucchiari et al., 2020, Italy [28, 30]	T1:E-cigarettes + LITC T2:Placebo + LITC	LITC		NR	12 W		continuous smoking abstinence/BV	Yes	6
Taylor et al., 2017, America [31]	individualized intervention	SC		15–20 Min/each	6 W		7-day-point prevalence/PRO, BV	NAE	3
Marshall et al., 2016, Australia [32]	individualized intervention	SC		NR	NR		7-day-point prevalence/PRO	NAE	12
Clark et al., 2004, America [33]	network resource	SC		NR	NR		7-day-point prevalence/PRO continuous smoking abstinence/PRO	NAE	12

The included studies are arranged in descending chronological order. “Masiero & Lucchiari” is the same study containing two reports from Masiero and Lucchiari. “Tremblay” is the same study containing two reports from Tremblay at 2019 and 2021

*T* Intervention group, *C* Control group, *M* Male, F Female, *yr* Year, *NR* Not reported, *SC* Standard care, *NAE* no adverse event, *W* Week, *Min* Minute, *M* Month, *LITC* Low-intensity telephone consultation, *PRO* Patient-reported outcome, *BV* Biochemical verification

^a^Fagestrom test for Nicotine Dependence score ≤ 5

^b^Fagestrom test for Nicotine Dependence score ≥ 6

^c^(Mean, SD)

^d^median (IQRF)

### Study quality assessment 

**Fig. 2:**
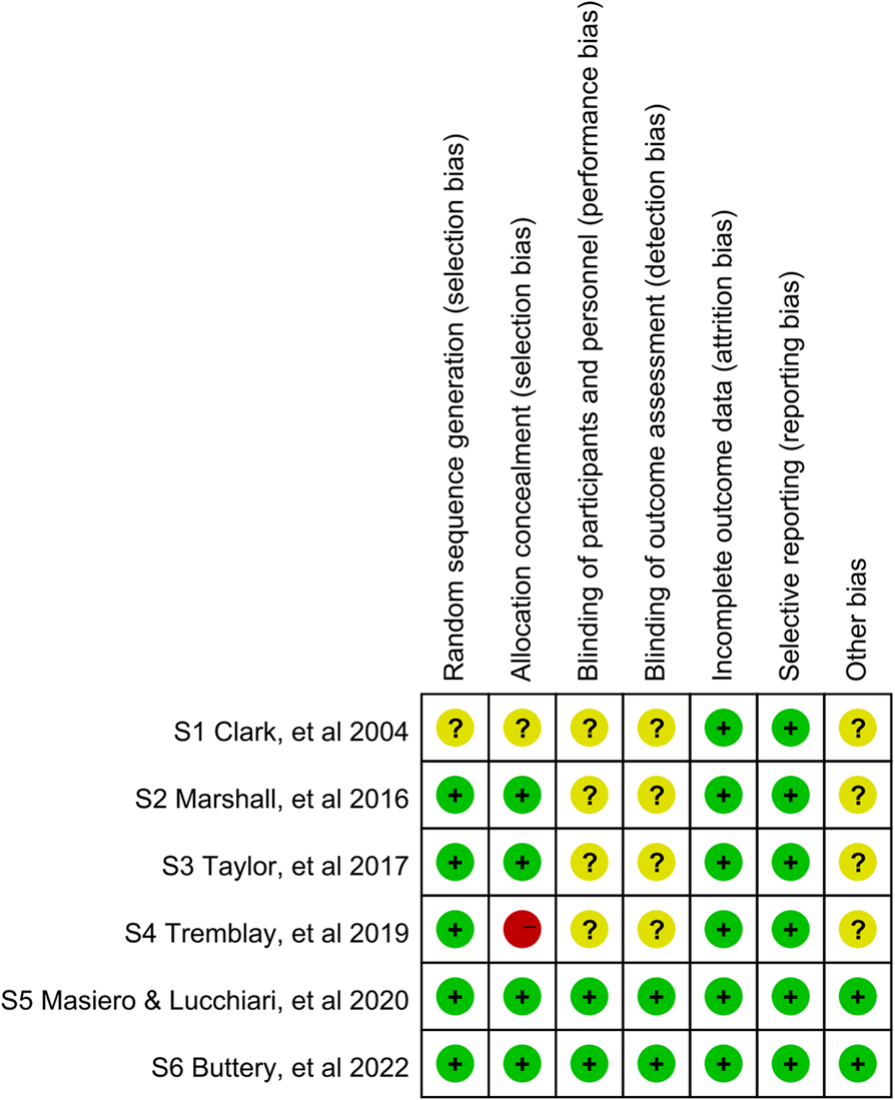
Methodological quality and bias risk assessment of the included trials in the effectiveness of smoking cessation on the high-risk population of lung cancer with early screening: a systematic review and meta analysis until January, 2022

The risk of bias in the included studies is shown in Fig. 2. All selected studies were randomized control studies. Five studies [26–32] explicitly reported the randomization method. One used a random number generator [32], one used the randomization list [28, 30], three used computerized randomization [26, 27, 29, 31], and the remaining one did not explicitly describe the randomization method [33]. Meanwhile, four studies reported that a special person was responsible for the distribution concealment of random numbers [26, 28, 30–32], one study failed to carry out distribution concealment [27, 29], and the remaining one study described insufficient information with an unclear risk of bias [33]. In addition, two studies indicated the blinding of participants and personnel together with the blinding of outcome assessments [26, 28, 30], the remaining four studies provided insufficient information [27, 29, 31–33]. In general, all studies presented a low risk of bias for selective outcome reporting and complete outcome data. Except for two studies [26, 28, 30], 4 out of 6 studies did not provide enough information for the evaluation of whether other biases were existing [27, 29, 31–33]. Publication bias was not determined because of the limited number of included studies (less than 10) for each comparison.

### Meta-analysis 

#### Primary outcome: 7-day-point prevalence of smoking abstinence

Five studies [26, 27, 29, 31–33] reported the effect of smoking cessation interventions on the 7-day-point prevalence of smoking abstinence by the patient-reported outcome. The results of outcomes were analyzed in subgroups according to different smoking cessation interventions and follow-up times, and the above five studies were included in both subgroups.

**Fig. 3:**
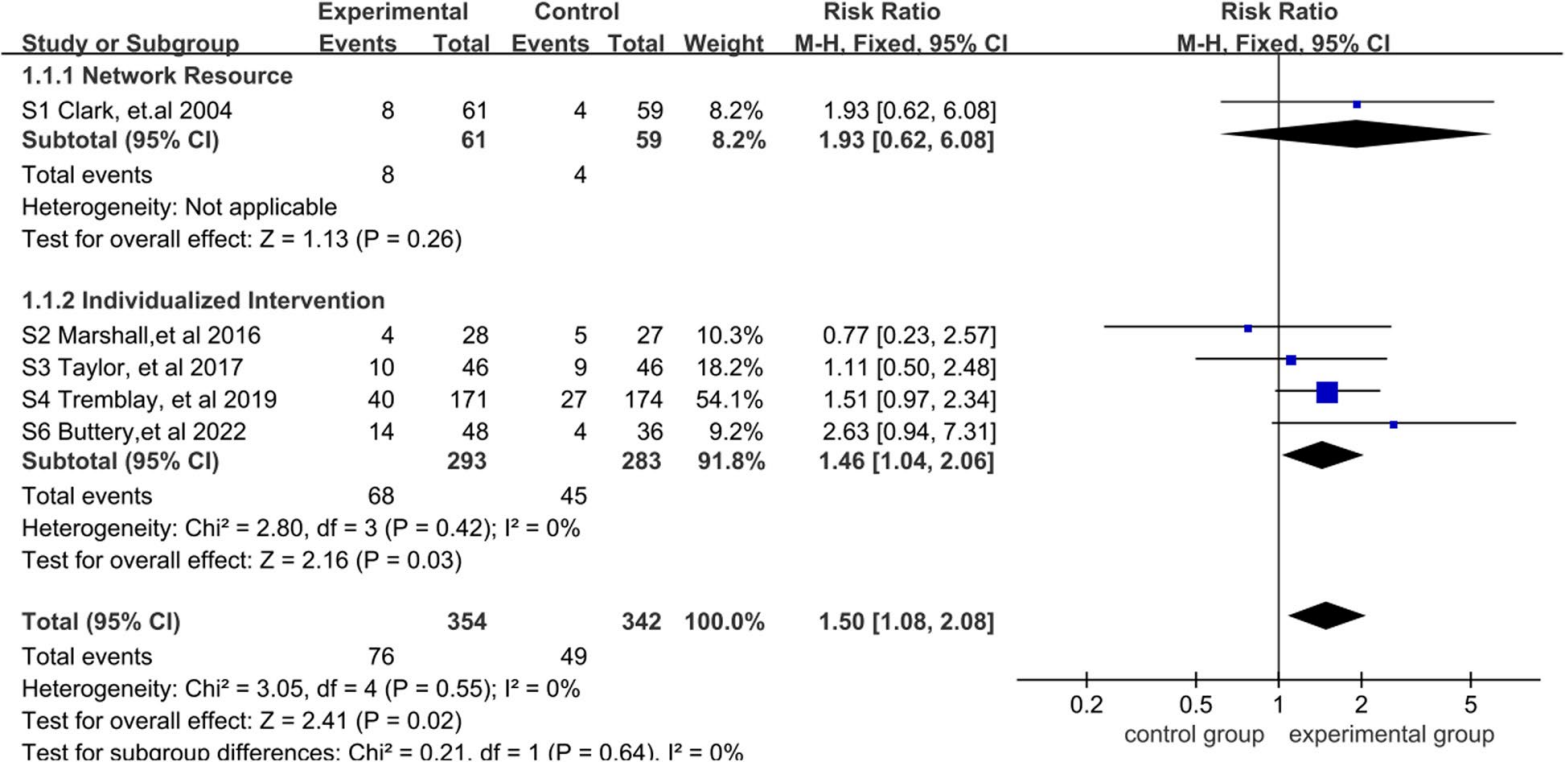
Meta-analysis and subgroup analysis result of 7-day-point prevalence smoking abstinence for the effectiveness of smoking cessation on the high-risk population of lung cancer with early screening: until January, 2022

The fixed-effects model was used for meta-analysis because low heterogeneity (*I*^2^ = 0%) was detected, and the results showed that the smoking cessation interventions group was significantly higher than the standard care group [RR = 1.50, 95% CI = (1.08, 2.08), *P* < 0.05]. This result indicated that smoking cessation intervention has a substantial influence on the high-risk population of lung cancer (*P* < 0.05). The results of subgroup analysis by smoking cessation interventions showed that there were no significant differences between network resources and standard care [RR = 1.93, 95% CI = (0.62, 6.08), *P* > 0.05]. However, the individualized intervention was considerably higher than that of standard care [RR = 1.46, 95% CI = (1.04,2.06), *P* < 0.05]. These results indicated that individualized intervention has a substantial effect on high-risk populations of lung cancer than standard care (*P* < 0.05). A more detailed forest plot is available in Fig. 3.

**Fig. 4:**
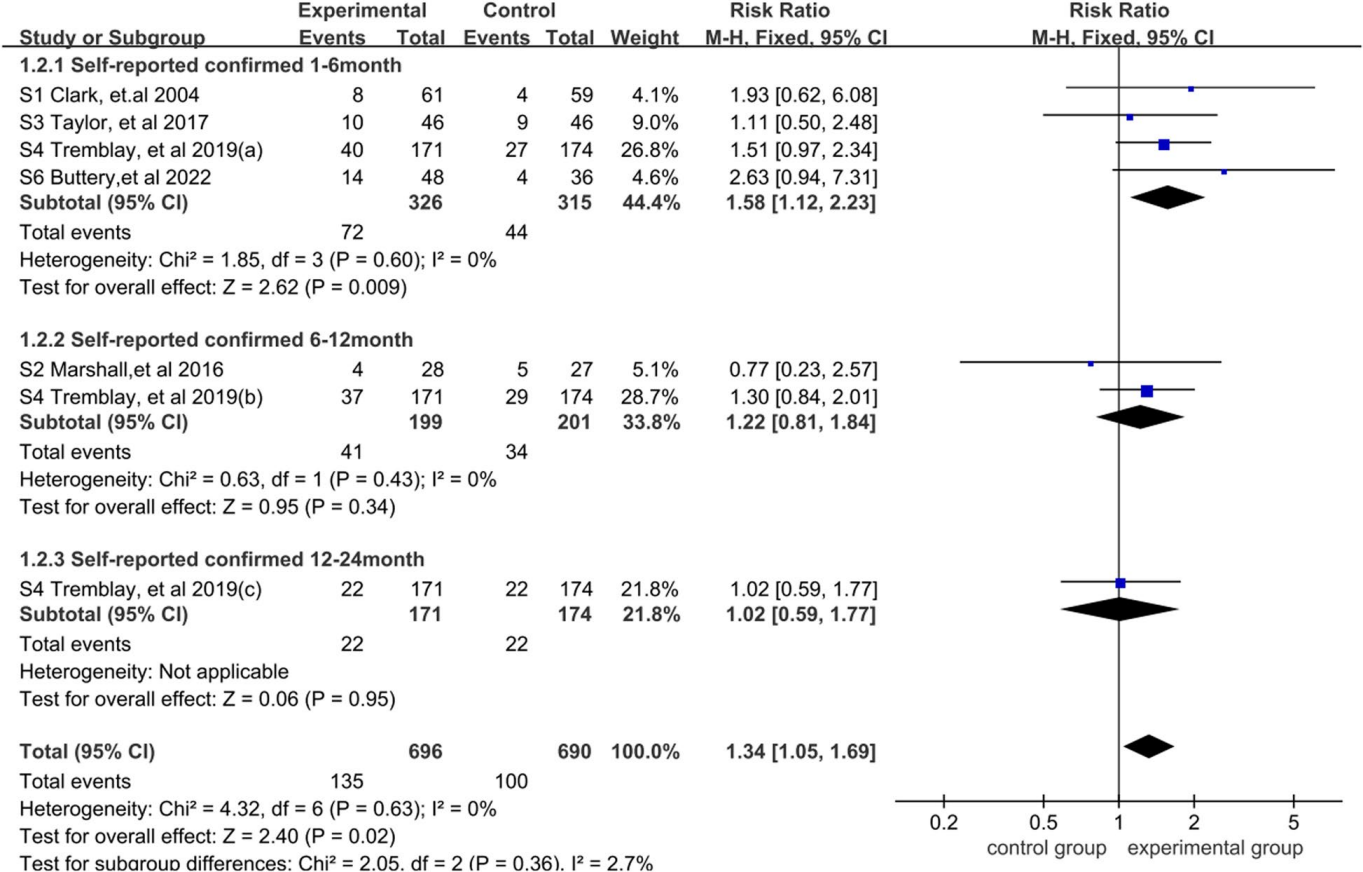
Meta-analysis and subgroup analysis result of 7-day-point prevalence smoking abstinence for the effectiveness of smoking cessation on the high-risk population of lung cancer with early screening: until January, 2022

According to the result of the subgroup divided by follow-up time in the patient-reported outcome, the fixed effects model was used for meta-analysis because of low heterogeneity (*I*^2^ = 0%). The results showed that the smoking cessation interventions group was considerably greater than the standard care group [RR = 1.34, 95% CI = (1.05, 1.69), *P* < 0.05], this result proved that smoking cessation intervention had a remarkable effect on the high-risk population of lung cancer (*P* < 0.05). The subgroup of follow-up time within 1–6 months showed that the smoking cessation interventions group was markedly higher than the standard care group [RR = 1.58, 95% CI = (1.12, 2.23), *P* < 0.05]. This result indicated that smoking cessation intervention within 1–6 months had a huge influence on the high-risk population of lung cancer (*P* < 0.05). While at 6–12 months [RR = 1.22, 95% CI = (0.81, 1.84), *P* > 0.05] and 12–24 months [RR = 1.02, 95% CI = (0.59, 1.77), *P* > 0.05], no significant difference between the two groups was observed. A more detailed forest plot is shown in Fig. 4.

#### Primary outcome: continuous smoking abstinence

Three studies [27–30, 33] reported the effect of smoking cessation interventions on continuous smoking abstinence. According to different smoking cessation interventions and follow-up times, the results of the outcomes were divided into subgroups, and a total of three studies were included in the smoking cessation interventions subgroups. The other two studies were divided into subgroups according to different follow-up times.

**Fig. 5:**
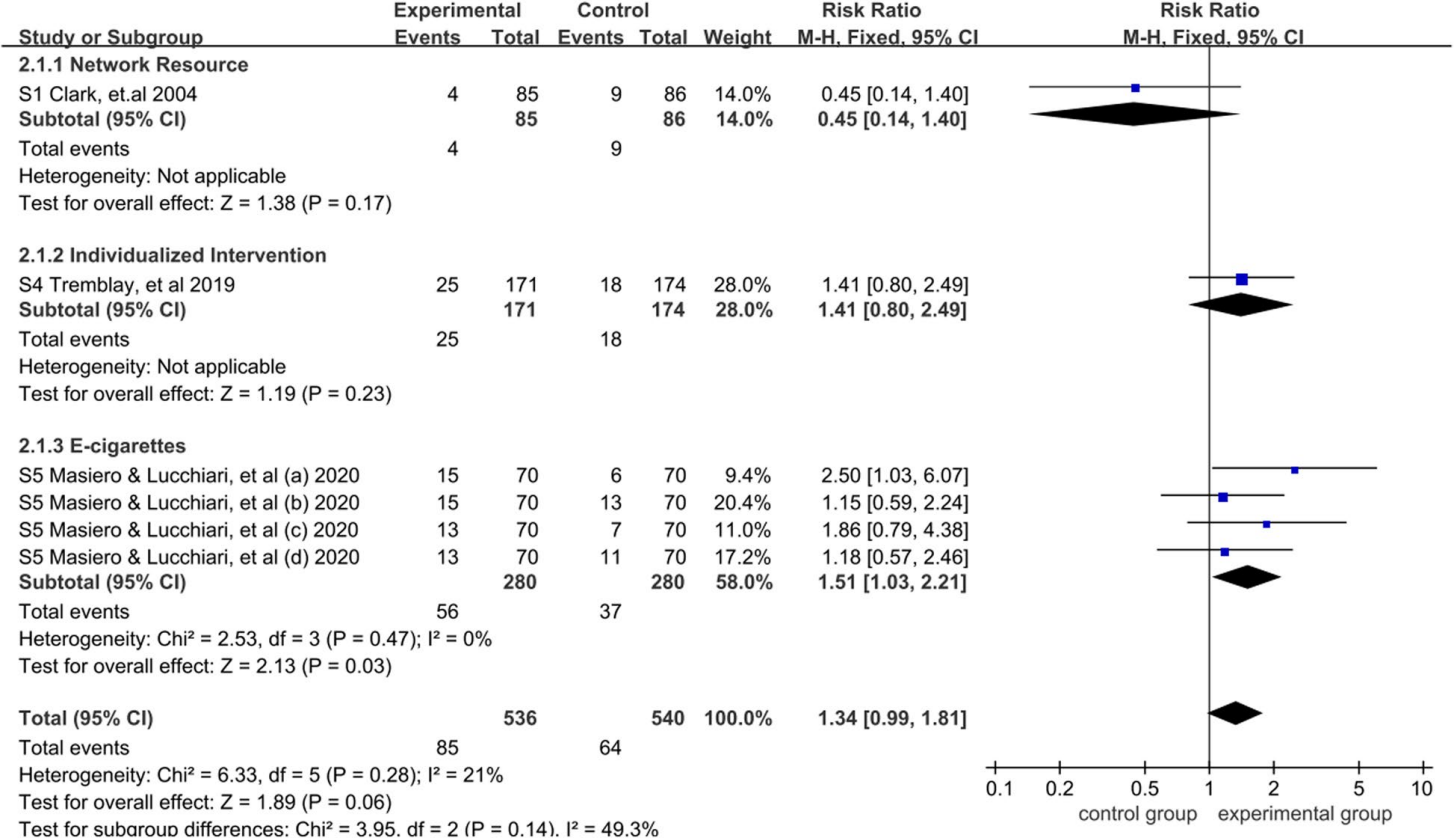
Meta-analysis and subgroup analysis result of continuous smoking abstinence for the effectiveness of smoking cessation on the high-risk population of lung cancer with early screening: until January, 2022

The fixed-effects model was used for meta-analysis because of low heterogeneity (*I*^2^ = 21%). There was no significant difference between smoking cessation interventions and standard care [RR = 1.34, 95% CI = (0.99,1.81), *P* > 0.05]. The results of subgroup analysis by smoking cessation interventions showed that E-cigarette [RR = 1.51, 95% CI = (1.03, 2.21), *P* < 0.05] was higher than that of the network resource [RR = 0.45, 95% CI = (0.14,1.40), *P* > 0.05] and individualized intervention [RR = 1.41, 95% CI = (0.80,2.94), *P* > 0.05]. These results indicated that E-cigarette had a determinate effect on high-risk population of lung cancer than standard care (*P* < 0.05). A more detailed forest plot is available in Fig. 5.

**Fig. 6:**
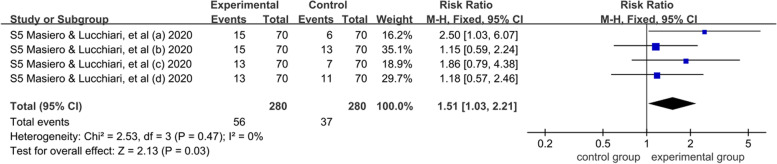
Meta-analysis results of continuous smoking abstinence for the effectiveness of smoking cessation on the high-risk population of lung cancer with early screening: a systematic review and meta analysis until January, 2022

According to the follow-up time in biochemical verification within 6 months, the fixed effects model was used for meta-analysis because of low heterogeneity (*I*^2^ = 0%). The results showed that within 1–6 months, the smoking cessation interventions group was significantly higher than the standard care group [RR = 1.51, 95% CI = (1.03, 2.21), *P* < 0.05], this result indicated that smoking cessation intervention within 1–6 months had a significant effect on the high-risk population of lung cancer (*P* < 0.05). A more detailed forest plot is available in Fig. 6. Furthermore, only one study [27, 29] reported biochemical verification at the 12-month follow-up (22/171 vs 19/174, *P* = 0.58). There was no significant difference in the continuous smoking cessation at the twelfth month between smoking cessation interventions and standard care.

In addition, one study [27, 29] reported the continuous smoking cessation rate verified by the patient-reported outcome. At the sixth-month follow-up (25/171 vs 18/174, *P* = 0.229), at the twelfth-month follow-up (24/171 vs 22/174, *P* = 0.704), and at the 24-month follow-up (36/168 vs 31/169, *P* = 0.48). There was no discernible difference in the rate of continuous smoking cessation between standard care and smoking cessation treatments.

#### Secondary outcome: adverse events

Out of the five included studies, only one study [28, 30] reported adverse events, such as burning throat, cough, nausea, headache, insomnia, stomachache, confusion, and dyspnea, which were caused by the usage of E-cigarettes. At the same time, placebo also reported the above adverse reactions, but the number was less than that of electronic cigarettes.

### Sensitivity analysis

In this study, sensitivity analysis was conducted for each outcome index by using sequential omission of each study. No significant changes in the effect size were found.

## Discussion

The results of the meta-analysis showed that in terms of the continuous smoking cessation rate, the smoking cessation effect of E-cigarettes was 1.51 times higher than that of standard care. In addition, the smoking cessation effect was statistically significant. In terms of the 7-day smoking cessation rate, the effect of individualized smoking cessation was 1.46 times greater than that of standard care, which improved the influence of smoking cessation. The results of the subgroup analysis of follow-up time showed that the smoking cessation impact of smoking cessation interventions within 6 months was better than that of standard care, not after 6 months.

The subjects of this systematic review are long-term smokers who undergo low-dose computed tomography, and the outcomes of this screening can help smokers become more aware of the negative effects of smoking and the advantages of quitting [34]. Smokers seeking low-dose computed tomography screening results are often at high risk of lung cancer, as stubborn smokers are highly dependent on nicotine and have a higher risk of lung cancer than ordinary smokers [34]. Some researchers [22] believed that the results of low-dose computed tomography screening can change the motivation of high-risk people to quit smoking, and the fear of lung cancer for high-risk groups can frequently become an important motivation for them to quit smoking [35]. The results of screening with low-dose computed tomography significantly influence the rate of smoking cessation. A study reported that [33] positive screening results can increase the quitting rate of smokers, while negative screening results may even reduce smokers’ awareness of cancer risk thus continuing to smoke. It is well known that smoking is not only a high-risk factor for lung cancer but also leads to other multisystem damage [30]. It is necessary to publicize the harmful effects of smoking and make smokers aware of the benefits of quitting smoking, especially for smokers whose screening results are negative. Unfortunately, the six studies included in this systematic review did not include the results of low-dose computed tomography screening as a factor affecting the rate of quitting smoking in the analysis. Future studies should consider the impact of low-dose computed tomography screening results on smoking cessation interventions, which can explore potential smoking cessation methods suitable for smokers with different low-dose computed tomography screening results to guide clinical research.

The smoking cessation interventions included in this systematic review include network resources, individualization and E-cigarettes. Among them, both network resources and individualization are categorized as psychological and behavioral intervention, and E-cigarettes are categorized as pharmacological approach [36]. The findings demonstrate that electronic cigarettes significantly affect smoking cessation. E-cigarettes are electronic devices that evaporate nicotine into aerosols for smokers to inhale. It provides smokers with sufficient levels of nicotine to help relieve withdrawal symptoms by simulating smoking behavior, vision, and perception [37]. Nicotine is an addictive substance in tobacco. Once smokers stop smoking, there will be a series of withdrawal symptoms, such as insomnia, irritability, inattention, fatigue, etc. [38]. Research shows that most people’s failure to quit smoking is due to abstinence reactions caused by a sudden interruption of nicotine in the process of quitting smoking [39]. Appropriate nicotine supplementation can reduce smokers’ mental dependence on nicotine, which in turn reduces nicotine craving and withdrawal symptoms. The results of this study show that E-cigarettes support people at high risk of lung cancer to quit smoking in the short term, which is consistent with the results of Harrell et. al [40]. However, despite their ability to aid in quitting smoking, e-cigarettes can cause unwanted respiratory side effects as cough, nauseousness, and dyspnea. Therefore, it is advised that clinical practice should make a wise decision to stop smoking based on thoroughly assessing its smoking cessation effect and negative effects. Because the study included in this systematic review only followed up on the effect of E-cigarettes for 6 months, therefore we were unable to determine the effect of e-cigarettes over the long term. Future research is hoped to follow up on the long-term effects of e-cigarettes and thus evaluate the long-term effects of quitting smoking.

Individual smoking cessation intervention aims at a series of tobacco dependence, smoking habit preference, psychological state of smoking cessation for smokers, and addressing the diverse demands of many sorts of smokers in terms of quitting smoking [26, 32]. Condoluci, et al. [9] declared that the individual smoking cessation interventions should be tailored according to the different smoking habits of smokers. The results of this systematic review shows that individual smoking cessation intervention is effective in the 7-day smoking cessation rate and ineffective in the continuous smoking cessation rate. This indicates that individual smoking cessation intervention can promote smoking cessation. The difference in the smoking cessation effect between network resources and standard care has not been found, although internet resources improve smokers’ desire for tobacco and control of smoking pleasure through social support [41]. For smokers who have smoked for at least 15 years, their physical and psychological dependence on nicotine is extremely high, and the withdrawal symptoms associated with quitting smoking are serious. A study found that [33] long-term smokers with a high risk of lung cancer. Their utilization rate of network resources is low, and the influence of network resources on smoking cessation drive and motivation of long-term lung cancer high-risk people is weak. It is suggested that future research strengthen social support for smoking cessation, such as through media publicity and public welfare activities. Improving the utilization rate of smoking cessation resources for smokers and then encouraging them to quit smoking.

The results of this systematic review subgroup analysis shows that the smoking cessation rate of smoking cessation interventions is significantly higher than that of standard care within 6 months of follow-up. However, the effect of smoking cessation interventions decreased gradually with the extension of follow-up time. Smokers who participated in low-dose computed tomography screening are at high risk of lung cancer, and participating in low-dose computed tomography screening itself has a warming effect on them. They may caution themselves to try their best to stop smoking and retain a comparatively high degree of recall and attention to smoking cessation therapies in a short period while getting smoking cessation intervention. This may be the reason why smoking cessation interventions are effective in quitting smoking in a short period. However, in reality, it can be challenging to continue their behavior of stopping smoking for an extended period. The gap between the intervention and measurement times is too wide, smokers’ memories of their interventions to stop smoking steadily deteriorate, and some smokers are unable to recall the occurrence of their interventions at all. With the extension of time, their shadow on low-dose computed tomography gradually weakened. At the same time, with the cumulative increase in withdrawal reaction caused by quitting smoking, they suffered great physical and psychological pain, which seriously affected the effect of smoking cessation interventions quitting smoking [42]. Our study observed that the effect of smoking cessation interventions on smokers’ memory and compliance to quitting smoking is limited by time. Therefore, it is suggested that the clinical implementation of smoking cessation interventions should be continuously strengthened and consolidated more intensively, especially after 6 months of intervention. Smokers’ internal driving force and motivation to quit smoking are gradually decreasing with the extension of time, and it is easy to give up psychologically. Because of smokers’ strong dependence on tobacco and forgetting about smoking cessation interventions, which makes the success rate of quitting smoking is lower in high-risk groups for lung cancer. Thus, it is necessary to increase the intensity of smoking cessation interventions or make appropriate adjustments for other more effective measures [10].

The present research revealed that in most studies, researchers mostly take the smoking cessation rate as the main outcome indicators [43, 44], such as the 7-day smoking cessation rate and the continuous smoking cessation rate. The 7-day quitting rate refers to the quitting rate within 7 days before the follow-up, and the continuous quitting rate refers to never smoking during the period from the date of quitting smoking to the end of the follow-up. The 7-day smoking cessation rate and continuous quitting rate are common end points for researchers to observe the short-term and/or long-term effects of smoking cessation interventions, which are important measurement standards for evaluating smoking cessation effectiveness[45]. During a given follow-up period, it can be considered that the continuous quitting rate is more stringent than the 7-day quitting rate [46]. Among the 6 studies included in this systematic review, only one article, “S4 Tremblay, et al.”, observed the continuous smoking cessation rate of smokers, which may be the reason for the ineffectiveness of individualized smoking cessation intervention in the continuous smoking cessation rate. These outcome indicators are evaluated by patient-reported outcomes or biochemical verification. patient-reported outcome refers to the research results directly reported by patients themselves. As an evaluation tool, it can directly and comprehensively reflect the true feelings of patients and contribute to patient-centered treatment [47]. Biochemical verification evaluates the results of quitting smoking by monitoring the level of carbon monoxide in smokers, which improves the science and rigor of the trial. The inclusion of biochemical verification in clinical research design can greatly improve the quality of the study [48]. However, only using patient-reported outcome or/(and) biochemical verification to evaluate the physiological changes of smokers, there is a certain level of limitation in the adoption of outcome indicators and the choice of evaluation methods, and the lack of observation of the psychological and behavioral changes of smokers, such as the change in smoking abstinence expectancies [49], motivation to quit smoking, the number of attempts to quit smoking and the degree of nicotine dependence, can be used as secondary outcome indicators of smoking cessation intervention. These data can also provide clues for improving smoking cessation interventions [11]. In addition, the degree and duration of smoking cessation interventions intervention were closely related to the effect of quitting smoking [50]. Among the 6 studies included in this systematic review, only one article, “S3 Taylor, et al.” [31] described the degree and duration of intervention, and the other, “S5 Masiero & Lucchiari, et al.” [28, 30] described the duration of intervention. Other studies have not reported the specific degree and time of smoking cessation intervention, which reduces the repeatability of the intervention and may also affect the judgment of the intervention effect in this study.

It is suggested that future studies fully report the research design, include a more detailed process and effect evaluation, and use a variety of methods to verify the effectiveness of the results to observe the smoking cessation effect of smoking cessation interventions more comprehensively.

### Limitations

There may be some limitations in this study. First, although the quality of the literature [26, 28, 30] in this study is high, it is still partly included in the poor quality of the articles, resulting in low quality of evidence potentially. Second, the literature we included was published in English. Studies published in other languages and studies that have been completed but not yet published may be omitted, resulting in publication and publication bias.

## Conclusions

In conclusion, the results of this systematic review show that smoking cessation intervention is effective for long-term lung cancer high-risk smokers who participate in early screening. Among them, E-cigarettes are the best, followed by individual smoking cessation. Future research should focus on improving the methodological quality of the trial, shortening the intervention period, and exploring more outcomes. Further studies should consider both the negative and positive influence of the low dose computed tomography screening population and report the test according to CONSORT guidelines; additionally, future research can also evaluate the smoking cessation effectiveness of smoking cessation interventions by observing changes in end points such as Fagestrom test, pack years, eCO, and spirometry; the smoking abstinence expectancies questionnaire (SAEQ) can be used to evaluate smoking abstinence expectancies [49]. This would help to explore smoking cessation interventions more comprehensively to provide scientific evidence of smoking cessation methods for long-term smokers at high risk of lung cancer.

## Supplementary Information

**Additional file 1.**

## Data Availability

The datasets used and analysed during the current study are available from the corresponding author on reasonable request.

## Abbreviations

RCT: Randomised controlled trials
Embase: Excerpta Medica Database
CENTRAL: Cochrane Central Register of Controlled Trials
CINAHL: Cumulative Index to Nursing and Allied Health Literature
PRISMA: The Preferred Reporting Items for Systematic Reviews and Meta-Analyses
RR: Relative risk
MD: Mean difference
SMD: Standardized mean difference
CI: Confidence Interval
T: Intervention group
C: Control group
M: Male
F: Female
yr: Year
SC: Standard care
NAE: No adverse event
NR: Not reported
W: Week
Min: Minute
M: Month
LITC: Low-intensity telephone consultation
PRO: Patient-reported outcome
BV: Biochemical verification
CONSORT: Consolidated Standards of Reporting Trials
