# Supplementary material for: Effectiveness of smoking cessation on the high-risk population of lung cancer with early screening: a systematic review and meta-analysis of randomized controlled trials until January 2022
Source: Arch Public Health. 2023 Jun 3;81:101. doi: 10.1186/s13690-023-01111-5 (PMC10239152; doi:10.1186/s13690-023-01111-5)
Supplement: Supplementary file 1 — Additional file 1. [file 13690_2023_1111_MOESM1_ESM.docx]

Additional file

# Search Strategy

## Proposed Databases (7 DataBases)

PubMed

EMBase

Web of Science

Cochrane Library (Cochrane Central Register of Controlled Trials, CENTRAL)

Cumulative Index to Nursing and Allied Health Literature (CINAHL)

PsycINFO

Science Direct

## Mesh Terms and Key Words

**1.2.1** [Mesh] Carcinoma, Non-Small-Cell Lung；Small Cell Lung Carcinoma；Lung Neoplasms； Adenocarcinoma of Lung；Adenocarcinoma, Bronchiolo-Alveolar；Bronchial Neoplasms；Carcinoma, Bronchogenic；Multiple Pulmonary Nodules；Pancoast Syndrome；Pulmonary Blastoma；Pulmonary Sclerosing Hemangioma

[Entry Term and Free Words]

| Carcinoma*, Non-Small-Cell Lung | non-small-cell lung* | Non small Cell Lung Ca* |
| --- | --- | --- |
| Carcinoma, Non-Small Cell Lung | Non-Small-Cell Lung Ca* | NSCLC |
| Carcinoma, Non Small Cell Lung | Lung Carcinoma*, Non-Small-Cell | Carcinoma, Large Cell |
| SCLC | small-cell lung cancer | Small cell lung cancer |
| Carcinoma, Small Cell | Small Cell Cancer Of The Lung | Carcinoma, Small Cell Lung |
| lung cancer* | Lung* tumo* | Lung carcinoma |
| Pulmonary Neoplasm* | Lung Neoplasm* | Cancer*, Lung |
| Neoplasm*, Lung | Neoplasm*, Pulmonary | Pulmonary Cancer* |
| Cancer*, Pulmonary | Cancer of the Lung | Cancer of Lung |
| pulm* tumo* | Lung Adenocarcinoma* | Adenocarcinoma*, Lung |
| Alveolar Adenocarcinoma* | Adenocarcinoma*, Bronchiolo Alveolar | Adenocarcinoma*, Alveolar |
| Bronchiolo-Alveolar Adenocarcinoma* | Bronchiolo-Alveolar Carcinoma* | Carcinoma*, Alveolar |
| Alveolar Carcinoma* | Alveolar Cell Carcinoma* | Carcinoma*, Alveolar Cell |
| Neoplasm*, Bronchial | Bronchial Neoplasm* | Bronch* Carcinoma* |
| Carcinoma*, Bronch* | Primary bronchogenic carcinoma | Multiple Pulmonary Nodule* |
| Pulmonary Nodule*, Multiple | Tumor, Pancoast | Syndrome, Pancoast* |
| Pancoast* Syndrome | Pancoast Tumor | Blastoma*, Pulmonary |
| Pulmonary Blastoma* | Pulmonary Sclerosing Hemangioma* | Sclerosing Hemangioma*, Pulmonary |
| Lung Sclerosing Hemangioma* | Sclerosing Hemangioma*, Lung | Hemangioma, Sclerosing, Pulmonary |
| Sclerosing Hemangioma of the Lung | lung* oat cell* | pulm* oat cell* |
| Oat Cell Lung Cancer | Oat Cell Carcinoma of Lung | lung* squamous |
| pulm* squamous | Carcinoma, Squamous Cell |  |

**1.2.2** [Mesh] Mass Screening；Early Detection of Cancer；Early Diagnosis；Multiphasic Screening；Mass Chest X-Ray；Tomography, Spiral Computed

[Entry Term and Free Words]

| Screening* | Mass Screening* | Screening*, Mass |
| --- | --- | --- |
| Cancer Early Detection | Screening, Cancer | Cancer Screening |
| Cancer Screening Test* | Screening Test*, Cancer | Test*, Cancer Screening |
| Early Diagnosis of Cancer | Cancer Early Diagnosis | lung cancer screening |
| Diagnosis, Early | Primary diagnosis | Early Detection of Disease |
| Disease Early Detection | diagnosis | Multiphasic Screening* |
| Screening*, Multiphasic | Automated Multiphasic Health Testing | Mass Chest X Ray |
| Mass Chest Xray* | Xray*, Mass Chest | Mass Chest X-Ray* |
| X-Ray*, Mass Chest | Scan*, Spiral CT | Spiral CT* |
| Helical CT* | CT*, Helical | CT,* Spiral |
| CT Scan*, Spiral | Scan*, Spiral CAT | Spiral CAT Scan* |
| Tomography, Spiral Computer* | Spiral Compute* Tomography | Helical Computed Tomography |
| Tomography, Helical Computed | Compute* Tomography, Spiral | CAT Scan*, Spiral |
| COSMOS | Continuous Observation of SMOking Subjects |  |

**1.2.3** [Mesh]Tobacco Use Disorder

[Entry Term and Free Words]

| Tobacco Use Disorder* | Disorder, Tobacco Use | Tobacco-Use Disorder |
| --- | --- | --- |
| Use Disorder, Nicotine | Disorder, Nicotine Use | Nicotine Use Disorder* |
| Disorder, Tobacco-Use | tobacco dependence* | Dependence, Tobacco |
| Dependence, Nicotine | Nicotine Dependence | continued smoking |
| current smok* | smoking status | still smok* |
| tobacco addict* | continued cigarette* | Cigarette smok* |
| Smoking | Smoker* | smok* post diagnos* |
| smok* postdiag* | smok* after diag* |  |

**1.2.4** [Mesh]Acupuncture；Anti-Anxiety Agents；Antidepressive Agents；Antidepressive Agents, Second-Generation；Azocines；Behavior Therapy；Benzazepines；Bupropion；Counseling；Directive Counseling；Exercise；Hypnosis；Nicotinic Agonists；Nicotinic Antagonists；Nortriptyline；Patient Education as Topic；Quinolizines；Quinoxalines；Smoking Cessation；Tobacco Use Cessation；Tobacco Use Cessation Devices; Electronic Nicotine Delivery Systems；Vaping

[Entry Term and Free Words]

| abstinen* | antidepressant* | fluoxetin* | imipramin* |
| --- | --- | --- | --- |
| anxiolyti* | Bupropion | cessation aid* | clonidine |
| cytisine | Dianicline | doxepin* | lazabemide* |
| lobeline | mecamylamine | moclobemide | nicobrevin* |
| nicotine gum* | nicotine inhaler* | nicotine nasal spray* | nicotine patch* |
| nicotine therap* | nicotine vaccin* | NRT | nicotine replacement therap* |
| paroxetin* | patient education | quit smoking | quitting smok* |
| rimonabant | selegilin* | sertralin* | silver acetat* |
| stop smoking | stopping smoking | tobacco cessation | tobacco treatment |
| tobacco use | tryptophan* | vareniclin* | venlafaxin* |
| zimeledin* |  |  |  |

**1.2.5** [Mesh] Randomized Controlled Trial; Controlled Clinical Trial

[Entry Term and Free Words]

| randomized | randomised | groups |
| --- | --- | --- |
| placebo | sham | "randomly |

## Searching Strategies

## 1.3.1 Pubmed 2022-01-26 (430 items)

**#1** ((((((((((Carcinoma, Non-Small-Cell Lung[MeSH Terms]) OR (Small Cell Lung Carcinoma[MeSH Terms])) OR (Lung Neoplasms[MeSH Terms])) OR (Adenocarcinoma of Lung[MeSH Terms])) OR (Adenocarcinoma, Bronchiolo-Alveolar[MeSH Terms])) OR (Bronchial Neoplasms[MeSH Terms])) OR (Carcinoma, Bronchogenic[MeSH Terms])) OR (Multiple Pulmonary Nodules[MeSH Terms])) OR (Pancoast Syndrome[MeSH Terms])) OR (Pulmonary Blastoma[MeSH Terms]))OR (Pulmonary Sclerosing Hemangioma[MeSH Terms]) Sort by: Best Match **254376**

**#2** ((((((((((((((((((((((((((((((((((((((((((((((((((((((((((((((((((((((((((Carcinoma*, Non-Small-Cell Lung[Title/Abstract]) OR (Small Cell Lung Carcinoma[Title/Abstract])) OR (Lung Neoplasm[Title/Abstract])) OR (Adenocarcinoma of Lung[Title/Abstract])) OR (Adenocarcinoma, bronchiole-Alveolar[Title/Abstract])) OR (Bronchial Neoplasm*[Title/Abstract])) OR (Carcinoma, Bronchogenic[Title/Abstract])) OR (Multiple Pulmonary Nodules[Title/Abstract])) OR (panacast Syndrome[Title/Abstract])) OR (Pulmonary Blastoma[Title/Abstract])) OR (Pulmonary Sclerosing Hemangioma[Title/Abstract])) OR (non-small-cell lung*[Title/Abstract])) OR (Non small Cell Lung Carcinoma*[Title/Abstract])) OR (Non small Cell Lung Cancer*[Title/Abstract])) OR (Carcinoma, Non-Small Cell Lung[Title/Abstract])) OR (Non-Small-Cell Lung Carcinoma*[Title/Abstract])) OR (Non-Small-Cell Lung Cancer*[Title/Abstract])) OR (NSCLC[Title/Abstract])) OR (Carcinoma, Non Small Cell Lung[Title/Abstract])) OR (Lung Carcinoma*, Non-Small-Cell[Title/Abstract])) OR (Carcinoma, Large Cell[Title/Abstract])) OR (SCLC[Title/Abstract])) OR (small-cell lung cancer[Title/Abstract])) OR (Small cell lung cancer[Title/Abstract])) OR (Carcinoma, Small Cell[Title/Abstract])) OR (Small Cell Cancer Of The Lung[Title/Abstract])) OR (Carcinoma, Small Cell Lung[Title/Abstract])) OR (lung cancer*[Title/Abstract])) OR (Lung* tumo*[Title/Abstract])) OR (Lung carcinoma[Title/Abstract])) OR (Pulmonary Neoplasm*[Title/Abstract])) OR (Cancer*, Lung[Title/Abstract])) OR (Neoplasm*, Lung[Title/Abstract])) OR (Neoplasm*, Pulmonary[Title/Abstract])) OR (Pulmonary Cancer*[Title/Abstract])) OR (Cancer*, Pulmonary[Title/Abstract])) OR (Cancer of the Lung[Title/Abstract])) OR (Cancer of Lung[Title/Abstract])) OR (pulm* tumo*[Title/Abstract])) OR (Lung Adenocarcinoma*[Title/Abstract])) OR (Adenocarcinoma*, Lung[Title/Abstract])) OR (Alveolar Adenocarcinoma*[Title/Abstract])) OR (Adenocarcinoma*, bronchiole Alveolar[Title/Abstract])) OR (Adenocarcinoma*, Alveolar[Title/Abstract])) OR (bronchiole-Alveolar Adenocarcinoma*[Title/Abstract])) OR (bronchiole-Alveolar Carcinoma*[Title/Abstract])) OR (Carcinoma*, Alveolar[Title/Abstract])) OR (Alveolar Carcinoma*[Title/Abstract])) OR (Alveolar Cell Carcinoma*[Title/Abstract])) OR (Carcinoma*, Alveolar Cell[Title/Abstract])) OR (Neoplasm*, Bronchial[Title/Abstract])) OR (Bronch* Carcinoma*[Title/Abstract])) OR (Carcinoma*, Bronch*[Title/Abstract])) OR (Primary bronchogenic carcinoma[Title/Abstract])) OR (Multiple Pulmonary Nodule*[Title/Abstract])) OR (Pulmonary Nodule*, Multiple[Title/Abstract])) OR (Tumor, pancoast[Title/Abstract])) OR (Syndrome, pancoast*[Title/Abstract])) OR (pancoast* Syndrome[Title/Abstract])) OR (pancoast Tumor[Title/Abstract])) OR (Blastoma*, Pulmonary[Title/Abstract])) OR (Pulmonary Blastoma*[Title/Abstract])) OR (Pulmonary Sclerosing Hemangioma*[Title/Abstract])) OR (Sclerosing Hemangioma*, Pulmonary[Title/Abstract])) OR (Lung Sclerosing Hemangioma*[Title/Abstract])) OR (Sclerosing Hemangioma*, Lung[Title/Abstract])) OR (Hemangioma, Sclerosing, Pulmonary[Title/Abstract])) OR (Sclerosing Hemangioma of the Lung[Title/Abstract])) OR (lung* oat cell*[Title/Abstract])) OR (pulm* oat cell*[Title/Abstract])) OR (Oat Cell Lung Cancer[Title/Abstract])) OR (Oat Cell Carcinoma of Lung[Title/Abstract])) OR (lung* squamous[Title/Abstract])) OR (pulm* squamous[Title/Abstract])) OR (Carcinoma, Squamous Cell[Title/Abstract]) Sort by: Best Match **1396790**

**#3 #1 OR #2** **1439174**

**#4** (((((Mass Screening[MeSH Terms]) OR (Early Detection of Cancer[MeSH Terms])) OR (Early Diagnosis[MeSH Terms])) OR (Multiphasic Screening[MeSH Terms])) OR (Mass Chest X-Ray[MeSH Terms])) OR (Tomography, Spiral Computed[MeSH Terms]) Sort by: Best Match **204249**

**#5** ((((((((((((((((((((((((((((((((((((((((((((((((Mass Screening*[Title/Abstract]) OR (Early Detection of Cancer[Title/Abstract])) OR (Early Diagnosis[Title/Abstract])) OR (Multiphasic Screening[Title/Abstract])) OR (Mass Chest X-Ray*[Title/Abstract])) OR (Tomography, Spiral Computed[Title/Abstract])) OR (Screening*[Title/Abstract])) OR (Screening*, Mass[Title/Abstract])) OR (Cancer Early Detection[Title/Abstract])) OR (Screening, Cancer[Title/Abstract])) OR (Cancer Screening[Title/Abstract])) OR (Cancer Screening Test*[Title/Abstract])) OR (Screening Test*, Cancer[Title/Abstract])) OR (Test*, Cancer Screening[Title/Abstract])) OR (Early Diagnosis of Cancer[Title/Abstract])) OR (Cancer Early Diagnosis[Title/Abstract])) OR (lung cancer screening[Title/Abstract])) OR (Diagnosis, Early[Title/Abstract])) OR (Primary diagnosis[Title/Abstract])) OR (Early Detection of Disease[Title/Abstract])) OR (Disease Early Detection[Title/Abstract])) OR (diagnosis[Title/Abstract])) OR (Multiphasic Screening*[Title/Abstract])) OR (Screening*, Multiphasic[Title/Abstract])) OR (Automated Multiphasic Health Testing[Title/Abstract])) OR (Mass Chest X Ray[Title/Abstract])) OR (Xray*, Mass Chest[Title/Abstract])) OR (Mass Chest XRay*[Title/Abstract])) OR (X-Ray*, Mass Chest[Title/Abstract])) OR (Scan*, Spiral CT[Title/Abstract])) OR (Spiral CT[Title/Abstract])) OR (Spiral CTs[Title/Abstract])) OR (Helical CT[Title/Abstract])) OR (Helical CTs[Title/Abstract])) OR (CT, Helical[Title/Abstract])) OR (CTs, Helical[Title/Abstract])) OR (CT, Spiral[Title/Abstract])) OR (CTs, Spiral[Title/Abstract])) OR (CT Scan*, Spiral[Title/Abstract])) OR (Scan*, Spiral CAT[Title/Abstract])) OR (Spiral CAT Scan*[Title/Abstract])) OR (Tomography, Spiral Computer*[Title/Abstract])) OR (Spiral Compute* Tomography[Title/Abstract])) OR (Helical Computed Tomography[Title/Abstract])) OR (Tomography, Helical Computed[Title/Abstract])) OR (Compute* Tomography, Spiral[Title/Abstract])) OR (CAT Scan*, Spiral[Title/Abstract])) OR (COSMOS[Title/Abstract])) OR (Continuous Observation of SMOking Subjects[Title/Abstract]) Sort by: Best Match **2305696**

**#6 #4 OR #5 2368439**

**#7** Tobacco Use Disorder[MeSH Terms] Sort by: Best Match **11902**

**#8** ((((((((((((((((((((((Tobacco Use Disorder*[Title/Abstract]) OR (Disorder, Tobacco Use[Title/Abstract])) OR (Tobacco-Use Disorder[Title/Abstract])) OR (Use Disorder, Nicotine[Title/Abstract])) OR (Disorder, Nicotine Use[Title/Abstract])) OR (Nicotine Use Disorder*[Title/Abstract])) OR (Disorder, Tobacco-Use[Title/Abstract])) OR (tobacco dependence*[Title/Abstract])) OR (Dependence, Tobacco[Title/Abstract])) OR (Dependence, Nicotine[Title/Abstract])) OR (Nicotine Dependence[Title/Abstract])) OR (continued smoking[Title/Abstract])) OR (current smok*[Title/Abstract])) OR (smoking status[Title/Abstract])) OR (still smok*[Title/Abstract])) OR (tobacco addict*[Title/Abstract])) OR (continued cigarette*[Title/Abstract])) OR (Cigarette smok*[Title/Abstract])) OR (Smoking[Title/Abstract])) OR (Smoker*[Title/Abstract])) OR (smok* post diagnos*[Title/Abstract])) OR (smok* postdiag*[Title/Abstract])) OR (smok* after diag*[Title/Abstract]) Sort by: Best Match **286318**

**#9 #7 OR #8 289008**

**#10** ((((((((((((((((((((((Acupuncture[MeSH Terms]) OR (Anti-Anxiety Agents[MeSH Terms])) OR (Antidepressive Agents[MeSH Terms])) OR (Antidepressive Agents, Second-Generation[MeSH Terms])) OR (Azocines[MeSH Terms])) OR (Behavior Therapy[MeSH Terms])) OR (Benzazepines[MeSH Terms])) OR (Bupropion[MeSH Terms])) OR (Counseling[MeSH Terms])) OR (Directive Counseling[MeSH Terms])) OR (Exercise[MeSH Terms])) OR (Hypnosis[MeSH Terms])) OR (Nicotinic Agonists[MeSH Terms])) OR (Nicotinic Antagonists[MeSH Terms])) OR (Nortriptyline[MeSH Terms])) OR (Patient Education as Topic[MeSH Terms])) OR (Quinolizines[MeSH Terms])) OR (Quinoxalines[MeSH Terms])) OR (Smoking Cessation[MeSH Terms])) OR (Tobacco Use Cessation[MeSH Terms])) OR (Tobacco Use Cessation Devices[MeSH Terms])) OR (Electronic Nicotine Delivery Systems[MeSH Terms])) OR (Vaping[MeSH Terms]) Sort by: Best Match **671143**

**#11** ((((((((((((((((((((((((((((((((((((((((((((((((((((((((((((((((Acupuncture[Title/Abstract]) OR (Anti-Anxiety Agents[Title/Abstract])) OR (Antidepressive Agents[Title/Abstract])) OR (Antidepressive Agents, Second-Generation[Title/Abstract])) OR (azonines[Title/Abstract])) OR (Behavior Therapy[Title/Abstract])) OR (Benzazepines[Title/Abstract])) OR (Bupropion[Title/Abstract])) OR (Counseling[Title/Abstract])) OR (Directive Counseling[Title/Abstract])) OR (Exercise[Title/Abstract])) OR (Hypnosis[Title/Abstract])) OR (Nicotinic Agonists[Title/Abstract])) OR (Nicotinic Antagonists[Title/Abstract])) OR (Nortriptyline[Title/Abstract])) OR (Patient Education as Topic[Title/Abstract])) OR (Quinolizines[Title/Abstract])) OR (Quinoxalines[Title/Abstract])) OR (Smoking Cessation[Title/Abstract])) OR (Tobacco Use Cessation[Title/Abstract])) OR (Tobacco Use Cessation Devices[Title/Abstract])) OR (Electronic Nicotine Delivery Systems[Title/Abstract])) OR (Vaping[Title/Abstract])) OR (abstinen*[Title/Abstract])) OR (antidepressant*[Title/Abstract])) OR (anxiolyti*[Title/Abstract])) OR (bupropion[Title/Abstract])) OR (cessation aid*[Title/Abstract])) OR (clonidine[Title/Abstract])) OR (cytisine[Title/Abstract])) OR (dianiline[Title/Abstract])) OR (doxepin*[Title/Abstract])) OR (fluoxetin*[Title/Abstract])) OR (imipramin*[Title/Abstract])) OR (lazabemide*[Title/Abstract])) OR (lobeline[Title/Abstract])) OR (mecamylamine[Title/Abstract])) OR (moclobemide[Title/Abstract])) OR (nicobrevin*[Title/Abstract])) OR (nicotine gum*[Title/Abstract])) OR (nicotine inhaler*[Title/Abstract])) OR (nicotine nasal spray*[Title/Abstract])) OR (nicotine patch*[Title/Abstract])) OR (nicotine therap*[Title/Abstract])) OR (nicotine vaccin*[Title/Abstract])) OR (NRT[Title/Abstract])) OR (nicotine replacement therap*[Title/Abstract])) OR (paroxetin*[Title/Abstract])) OR (patient education[Title/Abstract])) OR (quit smoking[Title/Abstract])) OR (quitting smok*[Title/Abstract])) OR (rimonabant[Title/Abstract])) OR (selegilin*[Title/Abstract])) OR (sertralin*[Title/Abstract])) OR (silver acetat*[Title/Abstract])) OR (smoking cessation[Title/Abstract])) OR (stop smoking[Title/Abstract])) OR (stopping smoking[Title/Abstract])) OR (tobacco cessation[Title/Abstract])) OR (tobacco treatment[Title/Abstract])) OR (tobacco use[Title/Abstract])) OR (tryptophan*[Title/Abstract])) OR (vareniclin*[Title/Abstract])) OR (venlafaxin*[Title/Abstract])) OR (zimeledin*[Title/Abstract]) Sort by: Best Match **674191**

**#12 #10 OR #11 1098198**

**#13 #3 AND #6 AND #9 AND #12 1396**

**#14** ((((((((randomized controlled trial[Publication Type]) OR (controlled clinical trial[Publication Type])) OR (randomized[Title/Abstract])) OR (randomised[Title/Abstract])) OR (placebo[Title/Abstract])) OR (sham[Title/Abstract])) OR (randomly[Title/Abstract])) OR ("trial[Title/Abstract])) OR (groups[Title/Abstract]) Sort by: Best Match **3468580**

**#15** ((animals[MeSH Terms]) NOT (humans[MeSH Terms])) AND (animals[MeSH Terms]) Sort by: Best Match **4948140**

**#16 #14 NOT #15 2954378**

**#17 #13 AND #16 430**

## 1.3.2 EMBase 2022-02-07 (310 items)

**#1** 'non small cell lung cancer'/exp OR 'lung tumor'/exp OR 'small cell lung cancer'/exp OR 'lung adenocarcinoma'/exp OR 'lung alveolus cell carcinoma'/exp OR 'lung carcinoma'/exp OR 'multiple pulmonary nodules'/exp OR 'pancoast tumor'/exp OR 'lung blastoma'/exp OR 'pulmonary sclerosing hemangioma'/exp **497455**

**#2** 'carcinoma*, non-small-cell lung':ab,ti OR 'small cell lung carcinoma':ab,ti OR 'lung neoplasm*':ab,ti OR 'adenocarcinoma of lung':ab,ti OR 'adenocarcinoma, bronchiole-alveolar':ab,ti OR 'bronchial neoplasm*':ab,ti OR 'carcinoma, bronchogenic':ab,ti OR 'multiple pulmonary nodules':ab,ti OR 'panacast syndrome':ab,ti OR 'pulmonary blastoma':ab,ti OR 'pulmonary sclerosing hemangioma':ab,ti OR 'non-small-cell lung*or non small cell lung carcinoma*':ab,ti OR 'non small cell lung cancer*':ab,ti OR 'carcinoma, non-small cell lung':ab,ti OR 'non-small-cell lung carcinoma*':ab,ti OR 'non-small-cell lung cancer*':ab,ti OR nsclc:ab,ti OR 'carcinoma, non small cell lung':ab,ti OR 'lung carcinoma*, non-small-cell':ab,ti OR 'carcinoma, large cell':ab,ti OR sclc:ab,ti OR 'small-cell lung cancer':ab,ti OR 'small cell lung cancer':ab,ti OR 'carcinoma, small cell':ab,ti OR 'small cell cancer of the lung':ab,ti OR 'carcinoma, small cell lung':ab,ti OR 'lung cancer*':ab,ti OR 'lung* tumo*':ab,ti OR 'lung carcinoma':ab,ti OR 'pulmonary neoplasm*':ab,ti OR 'cancer*, lung':ab,ti OR 'neoplasm*, lung':ab,ti OR 'neoplasm*, pulmonary':ab,ti OR 'pulmonary cancer*':ab,ti OR 'cancer*, pulmonary':ab,ti OR 'cancer of the lung':ab,ti OR 'cancer of lung':ab,ti OR 'pulm* tumo*':ab,ti OR 'lung adenocarcinoma*':ab,ti OR 'adenocarcinoma*, lung':ab,ti OR 'alveolar adenocarcinoma*':ab,ti OR 'adenocarcinoma*, bronchiole alveolar':ab,ti OR 'adenocarcinoma*, alveolar':ab,ti OR 'bronchiole-alveolar adenocarcinoma*':ab,ti OR 'bronchiole-alveolar carcinoma*':ab,ti OR 'carcinoma*, alveolar':ab,ti OR 'alveolar carcinoma*':ab,ti OR 'alveolar cell carcinoma*':ab,ti OR 'carcinoma*, alveolar cell':ab,ti OR 'neoplasm*, bronchial':ab,ti OR 'bronch* carcinoma*':ab,ti OR 'carcinoma*, bronch*':ab,ti OR 'primary bronchogenic carcinoma':ab,ti OR 'multiple pulmonary nodule*':ab,ti OR 'pulmonary nodule*, multiple':ab,ti OR 'tumor, pancoast':ab,ti OR 'syndrome, pancoast*':ab,ti OR 'pancoast* syndrome':ab,ti OR 'pancoast tumor':ab,ti OR 'blastoma*, pulmonary':ab,ti OR 'pulmonary blastoma*':ab,ti OR 'pulmonary sclerosing hemangioma*':ab,ti OR 'sclerosing hemangioma*, pulmonary':ab,ti OR 'lung sclerosing hemangioma*':ab,ti OR 'sclerosing hemangioma*, lung':ab,ti OR 'hemangioma, sclerosing, pulmonary':ab,ti OR 'sclerosing hemangioma of the lung':ab,ti OR 'lung* oat cell*':ab,ti OR 'pulm* oat cell*':ab,ti OR 'oat cell lung cancer':ab,ti OR 'oat cell carcinoma of lung':ab,ti OR 'lung* squamous':ab,ti OR 'pulm* squamous':ab,ti OR 'carcinoma, squamous cell':ab,ti **351450**

**#3 #1 OR #2 538339**

**#4** 'mass screening'/exp OR 'early cancer diagnosis'/exp OR 'early diagnosis'/exp OR 'multiphasic screening'/exp OR 'thorax radiography'/exp OR 'spiral computer assisted tomography'/exp **611273**

**#5** 'mass screening*':ab,ti OR 'early detection of cancer':ab,ti OR 'early diagnosis':ab,ti OR 'multiphasic screening':ab,ti OR 'mass chest x-ray*':ab,ti OR 'tomography, spiral computed':ab,ti OR screening*:ab,ti OR 'screening*, mass':ab,ti OR 'cancer early detection':ab,ti OR 'screening, cancer':ab,ti OR 'cancer screening':ab,ti OR 'cancer screening test*':ab,ti OR 'screening test*, cancer':ab,ti OR 'test*, cancer screening':ab,ti OR 'early diagnosis of cancer':ab,ti OR 'cancer early diagnosis':ab,ti OR 'lung cancer screening':ab,ti OR 'diagnosis, early':ab,ti OR 'primary diagnosis':ab,ti OR 'early detection of disease':ab,ti OR 'disease early detection':ab,ti OR diagnosis:ab,ti OR 'multiphasic screening*':ab,ti OR 'screening*, multiphasic':ab,ti OR 'automated multiphasic health testing':ab,ti OR 'mass chest x ray':ab,ti OR 'xray*, mass chest':ab,ti OR 'mass chest xray*':ab,ti OR 'x-ray*, mass chest':ab,ti OR 'scan*, spiral ct':ab,ti OR 'spiral ct':ab,ti OR 'spiral cts':ab,ti OR 'helical ct':ab,ti OR 'helical cts':ab,ti OR 'ct, helical':ab,ti OR 'cts, helical':ab,ti OR 'ct, spiral':ab,ti OR 'cts, spiral':ab,ti OR 'ct scan*, spiral':ab,ti OR 'scan*, spiral cat':ab,ti OR 'spiral cat scan*':ab,ti OR 'tomography, spiral computer*':ab,ti OR 'spiral compute* tomography':ab,ti OR 'helical computed tomography':ab,ti OR 'tomography, helical computed':ab,ti OR 'compute* tomography, spiral':ab,ti OR 'cat scan*, spiral':ab,ti OR cosmos:ab,ti OR 'continuous observation of smoking subjects':ab,ti **3207093**

**#6 #4 OR #5 3498560**

**#7** 'tobacco dependence'/exp **23248**

**#8** 'tobacco use disorder*':ab,ti OR 'disorder, tobacco use':ab,ti OR 'tobacco-use disorder':ab,ti OR 'use disorder, nicotine':ab,ti OR 'disorder, nicotine use':ab,ti OR 'nicotine use disorder*':ab,ti OR 'disorder, tobacco-use':ab,ti OR 'tobacco dependence*':ab,ti OR 'dependence, tobacco':ab,ti OR 'dependence, nicotine':ab,ti OR 'nicotine dependence':ab,ti OR 'continued smoking':ab,ti OR 'current smok*':ab,ti OR 'smoking status':ab,ti OR 'still smok*':ab,ti OR 'tobacco addict*':ab,ti OR 'continued cigarette*':ab,ti OR 'cigarette smok*':ab,ti OR 'smoking':ab,ti OR 'smoker':ab,ti OR 'smok* post diagnos*':ab,ti OR 'smok* postdiag*':ab,ti OR 'smok* after diag*':ab,ti **390449**

**#9 #7 OR #8 398811**

**#10** 'acupuncture'/exp OR 'anxiolytic agent'/exp OR 'antidepressant agent'/exp OR 'azocine derivative'/exp OR 'behavior therapy'/exp OR 'benzazepine derivative'/exp OR 'amfebutamone'/exp OR 'counseling'/exp OR 'directive counseling'/exp OR 'exercise'/exp OR 'hypnosis'/exp OR 'nicotinic agent'/exp OR 'nicotinic receptor blocking agent'/exp OR 'nortriptyline'/exp OR 'quinolizine derivative'/exp OR 'quinoxaline derivative'/exp OR 'smoking cessation'/exp OR 'nicotine gum'/exp OR 'electronic cigarette'/exp OR 'vaping'/exp **1474948**

**#11** acupuncture:ab,ti OR 'anti-anxiety agents':ab,ti OR 'antidepressive agents':ab,ti OR 'antidepressive agents, second-generation':ab,ti OR azonines:ab,ti OR 'behavior therapy':ab,ti OR benzazepines:ab,ti OR counseling:ab,ti OR 'directive counseling':ab,ti OR exercise:ab,ti OR hypnosis:ab,ti OR 'nicotinic agonists':ab,ti OR 'nicotinic antagonists':ab,ti OR nortriptyline:ab,ti OR 'patient education as topic':ab,ti OR quinolizines:ab,ti OR quinoxalines:ab,ti OR 'tobacco use cessation':ab,ti OR 'tobacco use cessation devices':ab,ti OR 'electronic nicotine delivery systems':ab,ti OR vaping:ab,ti OR abstinen*:ab,ti OR antidepressant*:ab,ti OR anxiolyti*:ab,ti OR bupropion:ab,ti OR 'cessation aid*':ab,ti OR clonidine:ab,ti OR cytisine:ab,ti OR dianiline:ab,ti OR doxepin*:ab,ti OR fluoxetin*:ab,ti OR imipramin*:ab,ti OR lazabemide*:ab,ti OR lobeline:ab,ti OR mecamylamine:ab,ti OR moclobemide:ab,ti OR nicobrevin*:ab,ti OR 'nicotine gum*':ab,ti OR 'nicotine inhaler*':ab,ti OR 'nicotine nasal spray*':ab,ti OR 'nicotine patch*':ab,ti OR 'nicotine therap*':ab,ti OR 'nicotine vaccin*':ab,ti OR nrt:ab,ti OR 'nicotine replacement therap*':ab,ti OR paroxetin*:ab,ti OR 'patient education':ab,ti OR 'quit smoking':ab,ti OR 'quitting smok*':ab,ti OR rimonabant:ab,ti OR selegilin*:ab,ti OR sertralin*:ab,ti OR 'silver acetat*':ab,ti OR 'smoking cessation':ab,ti OR 'stop smoking':ab,ti OR 'stopping smoking':ab,ti OR 'tobacco cessation':ab,ti OR 'tobacco treatment':ab,ti OR 'tobacco use':ab,ti OR tryptophan*:ab,ti OR vareniclin*:ab,ti OR venlafaxin*:ab,ti OR zimeledin*:ab,ti **885078**

**#12 #10 OR #11 1816831**

**#13** 'controlled clinical trial'/exp OR 'single blind procedure'/exp OR 'double blind procedure'/exp OR 'crossover procedure'/exp **934014**

**#14** random*:ab,ti OR crossover*:ab,ti OR 'cross near/3 over*':ab,ti OR placebo:ab,ti OR 'doubl* near/3 blind*':ab,ti OR 'doubl* near/3 mask*':ab,ti OR 'singl* near/3 blind*':ab,ti OR 'singl* near/3 mask*':ab,ti OR 'trebl* near/3 blind*':ab,ti OR 'trebl* near/3 mask*':ab,ti OR 'tripl* near/3 blind*':ab,ti OR 'tripl* near/3 mask*':ab,ti OR assign*:ab,ti OR allocat*:ab,ti OR volunteer*:ab,ti **2460826**

**#15 #13 OR #14 2711306**

**#16 #3 AND #6 AND #9 AND #12 AND #15 310**

## 1.3.3 Web of Science 2022-02-07 (670 items)

**#1** TOPIC:(((((((((((((((((((((((((((((((((((((((((((((((((((((((((((((((((((((((((Carcinoma*, Non-Small-Cell Lung OR Small Cell Lung Carcinoma) OR Lung Neoplasm*) OR Adenocarcinoma of Lung) OR Adenocarcinoma, bronchiole-Alveolar) OR Bronchial Neoplasm*) OR Carcinoma, Bronchogenic) OR Multiple Pulmonary Nodules) OR panocast Syndrome) OR Pulmonary Blastoma) OR Pulmonary Sclerosing Hemangioma) OR non-small-cell lung*OR Non small Cell Lung Carcinoma*) OR Non small Cell Lung Cancer*) OR Carcinoma, Non-Small Cell Lung) OR Non-Small-Cell Lung Carcinoma*) OR Non-Small-Cell Lung Cancer*) OR NSCLC) OR Carcinoma, Non Small Cell Lung) OR Lung Carcinoma*, Non-Small-Cell) OR Carcinoma, Large Cell) OR SCLC) OR small-cell lung cancer) OR Small cell lung cancer) OR Carcinoma, Small Cell) OR Small Cell Cancer Of The Lung) OR Carcinoma, Small Cell Lung) OR lung cancer*) OR Lung* tumo*) OR Lung carcinoma) OR Pulmonary Neoplasm*) OR Cancer*, Lung) OR Neoplasm*, Lung) OR Neoplasm*, Pulmonary) OR Pulmonary Cancer*) OR Cancer*, Pulmonary) OR Cancer of the Lung) OR Cancer of Lung) OR pulm* tumo*) OR Lung Adenocarcinoma*) OR Adenocarcinoma*, Lung) OR Alveolar Adenocarcinoma*) OR Adenocarcinoma*, bronchiole Alveolar) OR Adenocarcinoma*, Alveolar) OR bronchiole-Alveolar Adenocarcinoma*) OR bronchiole-Alveolar Carcinoma*) OR Carcinoma*, Alveolar) OR Alveolar Carcinoma*) OR Alveolar Cell Carcinoma*) OR Carcinoma*, Alveolar Cell) OR Neoplasm*, Bronchial) OR Bronch* Carcinoma*) OR Carcinoma*, Bronch*) OR Primary bronchogenic carcinoma) OR Multiple Pulmonary Nodule*) OR Pulmonary Nodule*, Multiple) OR Tumor, pancras) OR Syndrome, pancras*) OR pancras* Syndrome) OR pancras Tumor) OR Blastoma*, Pulmonary) OR Pulmonary Blastoma*) OR Pulmonary Sclerosing Hemangioma*) OR Sclerosing Hemangioma*, Pulmonary) OR Lung Sclerosing Hemangioma*) OR Sclerosing Hemangioma*, Lung) OR Hemangioma, Sclerosing, Pulmonary) OR Sclerosing Hemangioma of the Lung) OR lung* oat cell*) OR pulm* oat cell*) OR Oat Cell Lung Cancer) OR Oat Cell Carcinoma of Lung) OR lung* squamous) OR pulm* squamous) OR Carcinoma, Squamous Cell) (Topic) **1156868**

**#2** TOPIC：((((((((((((((((((((((((((((((((((((((((((((((((Mass Screening* OR Early Detection of Cancer) OR Early Diagnosis) OR Multiphasic Screening) OR Mass Chest X-Ray*) OR Tomography, Spiral Computed) OR Screening*) OR Screening*, Mass) OR Cancer Early Detection) OR Screening, Cancer) OR Cancer Screening) OR Cancer Screening Test*) OR Screening Test*, Cancer) OR Test*, Cancer Screening) OR Early Diagnosis of Cancer) OR Cancer Early Diagnosis) OR lung cancer screening) OR Diagnosis, Early) OR Primary diagnosis) OR Early Detection of Disease) OR Disease Early Detection) OR diagnosis) OR Multiphasic Screening*) OR Screening*, Multiphasic) OR Automated Multiphasic Health Testing) OR Mass Chest X Ray) OR Xray*, Mass Chest) OR Mass Chest XRay*) OR X-Ray*, Mass Chest) OR Scan*, Spiral CT) OR Spiral CT) OR Spiral CTs) OR Helical CT) OR Helical CTs) OR CT, Helical) OR CTs, Helical) OR CT, Spiral) OR CTs, Spiral) OR CT Scan*, Spiral) OR Scan*, Spiral CAT) OR Spiral CAT Scan*) OR Tomography, Spiral Computer*) OR Spiral Compute* Tomography) OR Helical Computed Tomography) OR Tomography, Helical Computed) OR Compute* Tomography, Spiral) OR CAT Scan*, Spiral) OR COSMOS) OR Continuous Observation of SMOking Subjects) **6392976**

**#3** TOPIC:((((((((((((((((((((((Tobacco Use Disorder* OR Disorder, Tobacco Use) OR Tobacco-Use Disorder) OR Use Disorder, Nicotine) OR Disorder, Nicotine Use) OR Nicotine Use Disorder*) OR Disorder, Tobacco-Use) OR tobacco dependence*) OR Dependence, Tobacco) OR Dependence, Nicotine) OR Nicotine Dependence) OR continued smoking) OR current smok*) OR smoking status) OR still smok*) OR tobacco addict*) OR continued cigarette*) OR Cigarette smok*) OR Smoking ) OR Smoker*) OR smok* post diagnos*) OR smok* postdiag*) OR smok* after diag*) **695501**

**#4** TOPIC:((((((((((((((((((((((((((((((((((((((((((((((((((((((((((((((((Acupuncture OR Anti-Anxiety Agents) OR Antidepressive Agents) OR Antidepressive Agents, Second-Generation) OR azonines) OR Behavior Therapy) OR Benzazepines) OR Bupropion) OR Counseling) OR Directive Counseling) OR Exercise) OR Hypnosis) OR Nicotinic Agonists) OR Nicotinic Antagonists) OR Nortriptyline) OR Patient Education as Topic) OR Quinolizines) OR Quinoxalines) OR Smoking Cessation) OR Tobacco Use Cessation) OR Tobacco Use Cessation Devices) OR Electronic Nicotine Delivery Systems) OR Vaping) OR abstinen*) OR antidepressant*) OR anxiolyti*) OR bupropion) OR cessation aid*) OR clonidine) OR cytisine) OR dianiline) OR doxepin*) OR fluoxetin*) OR imipramin*) OR lazabemide*) OR lobeline) OR mecamylamine) OR moclobemide) OR nicobrevin*) OR nicotine gum*) OR nicotine inhaler*) OR nicotine nasal spray*) OR nicotine patch*) OR nicotine therap*) OR nicotine vaccin*) OR NRT) OR nicotine replacement therap*) OR paroxetin*) OR patient education) OR quit smoking) OR quitting smok*) OR rimonabant) OR selegilin*) OR sertralin*) OR silver acetat*) OR smoking cessation) OR stop smoking) OR stopping smoking) OR tobacco cessation) OR tobacco treatment) OR tobacco use) OR tryptophan*) OR vareniclin*) OR venlafaxin*) OR zimeledin*) **3129845**

**#5 #1 AND #2 AND #3 AND #4 6323**

**#6** random* (Topic) **2714713**

**#7 #5 AND #6 670**

## 1.3.4 Cochrane Library (Cochrane Central Register of Controlled Trials, CENTRAL) 2022-02-07 (485 items)

**#1** MeSH descriptor: [Carcinoma, Non-Small-Cell Lung] explode all trees **4647**

**#2** MeSH descriptor: [Lung Neoplasms] explode all trees **8309**

**#3** MeSH descriptor: [Small Cell Lung Carcinoma] explode all trees **444**

**#4** MeSH descriptor: [Adenocarcinoma of Lung] explode all trees **114**

**#5** MeSH descriptor: [Adenocarcinoma, Bronchiolo-Alveolar] explode all trees **40**

**#6** MeSH descriptor: [Bronchial Neoplasms] explode all trees **5285**

**#7** MeSH descriptor: [Carcinoma, Bronchogenic] explode all trees **5178**

**#8** MeSH descriptor: [Multiple Pulmonary Nodules] explode all trees **55**

**#9** MeSH descriptor: [Pancoast Syndrome] explode all trees **2**

**#10** MeSH descriptor: [Pulmonary Blastoma] explode all trees **0**

**#11** (Carcinoma*, Non-Small-Cell Lung OR Lung Neoplasms OR Small Cell Lung Carcinoma OR Adenocarcinoma of Lung OR Adenocarcinoma, Bronchiolo-Alveolar OR Bronchial Neoplasms OR Carcinoma, Bronchogenic OR Multiple Pulmonary Nodules OR Pancoast Syndrome OR Pulmonary Blastoma OR Pulmonary Sclerosing Hemangioma OR lung cancer* OR lung tumo* OR non-small-cell lung* OR NSCLC OR SCLC OR small-cell lung cancer OR bronchogenic carcinoma OR Primary bronchogenic carcinoma OR Lung carcinoma OR Small cell lung cancer OR Carcinoma, Non Small Cell Lung OR Lung Carcinoma*, Non-Small-Cell OR Non-Small-Cell Lung Cancer* OR Lung Carcinoma* OR Non small Cell Lung Cancer OR Non Small Cell Lung Carcinoma OR Carcinoma, Non-Small Cell Lung OR Pulmonary Neoplasms OR Neoplasm*, Lung OR Lung Neoplasm OR Neoplasm*, Pulmonary OR Pulmonary Neoplasm OR Cancer*, Lung OR Pulmonary Cancer* OR Cancer*, Pulmonary OR Cancer of the Lung OR Cancer of Lung OR Small Cell Lung Cancer OR Oat Cell Lung Cancer OR Small Cell Cancer Of The Lung OR Carcinoma, Small Cell Lung OR Oat Cell Carcinoma of Lung OR Lung Adenocarcinoma* OR Adenocarcinoma*, Lung OR Adenocarcinoma*, Bronchiolo Alveolar OR Bronchiolo-Alveolar Adenocarcinoma* OR Adenocarcinoma*, Alveolar OR Alveolar Adenocarcinoma* OR Carcinoma*, Alveolar OR Alveolar Carcinoma* OR Bronchiolo-Alveolar Carcinoma* OR Alveolar Cell Carcinoma* OR Carcinoma*, Alveolar Cell OR Bronchiolar Carcinoma* OR Carcinoma*, Bronchiol* OR Bronch* Carcinoma* OR Neoplasm*, Bronchial OR Bronchial Neoplasm OR Carcinoma*, Bronch* OR Bronchial Carcinoma* OR Multiple Pulmonary Nodule OR Pulmonary Nodule*, Multiple OR Syndrome, Pancoast* OR Pancoast* Syndrome OR Pancoast Tumor OR Tumor, Pancoast OR Blastoma*, Pulmonary OR Pulmonary Blastomas OR Pulmonary Sclerosing Hemangiomas OR Sclerosing Hemangioma*, Pulmonary OR Lung Sclerosing Hemangioma* OR Sclerosing Hemangioma*, Lung OR Hemangioma, Sclerosing, Pulmonary OR Sclerosing Hemangioma of the Lung OR Carcinoma, Squamous Cell OR Carcinoma, Large Cell OR Carcinoma, Small Cell):ti,ab,kw **39429**

**#12 #1 OR #2 OR #3 OR #4 OR #5 OR #6 OR #7 OR #8 OR #9 OR #10 OR #11 39434**

**#13** MeSH descriptor: [Mass Screening] explode all trees **4019**

**#14** MeSH descriptor: [Early Detection of Cancer] explode all trees **1384**

**#15** MeSH descriptor: [Early Diagnosis] explode all trees **1940**

**#16** MeSH descriptor: [Multiphasic Screening] explode all trees **16**

**#17** MeSH descriptor: [Mass Chest X-Ray] explode all trees **22**

**#18** MeSH descriptor: [Tomography, Spiral Computed] explode all trees **335**

**#19** (Screen* OR Diagnosis OR Detection OR Test* OR Compute* Tomography OR CT OR ray):ti,ab,kw **628067**

**#20 #13 OR #14 OR #15 OR #16 OR #17 OR #18 OR #19 628067**

**#21** (( lung* OR pulm*) AND (tumo* OR Squamous OR oat cell*)):ti,ab,kw **13089**

**#22 #12 OR #21 39869**

**#23** MeSH descriptor: [Tobacco Use Disorder] explode all trees **1768**

**#24 (**Tobacco OR Cigarette OR Smok* OR Nicotine):ti,ab,kw **42759**

**#25** (smok* AND (post diagnos* OR postdiag* OR after diag*)):ti,ab,kw **3397**

**#26** **#23 OR #24 OR #25 42759**
**#27** MeSH descriptor: [Acupuncture] explode all trees **303**

**#28** MeSH descriptor: [Anti-Anxiety Agents] explode all trees **2211**

**#29** MeSH descriptor: [Antidepressive Agents] explode all trees **5989**

**#30** MeSH descriptor: [Antidepressive Agents, Second-Generation] explode all trees **1356**

**#31** MeSH descriptor: [Azocines] explode all trees **177**

**#32** MeSH descriptor: [Behavior Therapy] explode all trees **18165**

**#33** MeSH descriptor: [Benzazepines] explode all trees **12439**

**#34** MeSH descriptor: [Bupropion] explode all trees **851**

**#35** MeSH descriptor: [Counseling] explode all trees **5836**

**#36** MeSH descriptor: [Exercise] explode all trees **27342**

**#37** MeSH descriptor: [Hypnosis] explode all trees **755**

**#38** MeSH descriptor: [Nicotinic Agonists] explode all trees **754**

**#39** MeSH descriptor: [Nicotinic Antagonists] explode all trees **45**

**#40** MeSH descriptor: [Nortriptyline] explode all trees **451**

**#41** MeSH descriptor: [Patient Education as Topic] explode all trees **9221**

**#42** MeSH descriptor: [Quinolizines] explode all trees **242**

**#43** MeSH descriptor: [Quinoxalines] explode all trees **1018**

**#44** MeSH descriptor: [Smoking Cessation] explode all trees **4321**

**#45** MeSH descriptor: [Tobacco Use Cessation] explode all trees **120**

**#46** MeSH descriptor: [Tobacco Use Cessation Devices] explode all trees **600**

**#47** (Acupuncture OR Anti-anxiety Agents OR Antidepressive Agents OR Antidepressive Agents, Second-Generation OR Azocines OR Behavior Therapy OR Benzazepines OR Bupropion OR Counseling OR Directive Counseling OR Exercise OR Hypnosis OR Nicotinic Agonists OR Nicotinic Antagonists OR Nortriptyline OR Patient Education as Topic OR Quinolizines OR Quinoxalines OR Smoking Cessation OR Tobacco Use Cessation OR Tobacco Use Cessation Products OR Acupuncture OR Anti-Anxiety Agents OR Antidepressive Agents OR Antidepressive Agents, Second-Generation OR Azocines OR Behavior Therapy OR Benzazepines OR Bupropion OR Counseling OR Directive Counseling OR Exercise OR Hypnosis OR Nicotinic Agonists OR Nicotinic Antagonists OR Nortriptyline* OR Patient Education as Topic OR Quinolizines OR Smoking Cessation OR Tobacco Use Cessation OR Tobacco Use Cessation Products OR abstinen* OR acupuncture OR antidepressant* OR anxiolyti* OR bupropion OR cessation aid* OR clonidine OR counseling OR cytisine OR dianicline OR doxepin* OR exercise OR fluoxetin* OR hypnosis OR imipramin* OR lazabemide* OR lobeline OR mecamylamine OR moclobemide OR nicobrevin* OR nicotine gum* OR nicotine inhaler* OR nicotine nasal spray* OR nicotine patch* OR nicotine therap* OR nicotine vaccin* OR Nortriptyline OR NRT nicotine replacement therap* OR paroxetin* OR patient education OR quit smoking OR quitting smok* OR rimonabant OR selegilin* OR sertralin* OR silver acetat* OR smoking cessation OR stop smoking OR stopping smoking OR tobacco cessation OR tobacco treatment OR tobacco use OR tryptophan* OR vareniclin* OR venlafaxin* OR zimeledin*):ti,ab,kw **243181**

**#48 #27 OR #28 OR #29 OR #30 OR #31 OR #32 OR #33 OR #34 OR #35 OR #36OR #37 OR #38 OR #39 OR #40 OR #41 OR #42 OR #43 OR #44 OR #45 OR #46OR #47 262586**

**#49** MeSH descriptor: [Randomized Controlled Trial] explode all trees **119**

**#50** MeSH descriptor: [Controlled Clinical Trial] explode all trees **128**

**#51** (“randomized controlled trial” OR “controlled clinical trial” OR randomized OR randomised OR placebo OR sham OR randomly OR trial OR groups):ti,ab,kw **1356455**

**#52 #49 OR #50 OR #51 1356455**

**#53** (animal):ti,ab,kw **19437**

**#54 #52 NOT #53 1339294**

**#55 #20 AND #22 AND #26 AND #48 AND #54 485**

## 1.3.5 Cumulative Index to Nursing and Allied Health Literature (CINAHL) 2022-02-07(53 items)

**#1** MM Carcinoma, Non-Small-Cell Lung OR MM Small Cell Lung Carcinoma OR MM Lung Neoplasms OR MM Adenocarcinoma of Lung OR MM Adenocarcinoma, Bronchiolo-Alveolar OR MM Bronchial Neoplasms OR MM Carcinoma, Bronchogenic OR MM Carcinoma, Bronchogenic OR MM Pancoast Syndrome OR MM Pulmonary Blastoma OR MM Pulmonary Sclerosing Hemangioma **39822**

**#2** TI Carcinoma*, Non-Small-Cell Lung or Lung Neoplasms or Small Cell Lung Carcinoma or Adenocarcinoma of Lung or Adenocarcinoma, Bronchiolo-Alveolar or Bronchial Neoplasms or Carcinoma, Bronchogenic or Multiple Pulmonary Nodules or Pancoast Syndrome or Pulmonary Blastoma or Pulmonary Sclerosing Hemangioma or lung cancer* or lung tumo* or non-small-cell lung* or NSCLC or SCLC or small-cell lung cancer or bronchogenic carcinoma or Primary bronchogenic carcinoma or Lung carcinoma or Small cell lung cancer or Carcinoma, Non Small Cell Lung or Lung Carcinoma*, Non-Small-Cell or Non-Small-Cell Lung Cancer* or Lung Carcinoma* or Non small Cell Lung Cancer or Non Small Cell Lung Carcinoma or Carcinoma, Non-Small Cell Lung or Pulmonary Neoplasms or Neoplasm*, Lung or Lung Neoplasm or Neoplasm*, Pulmonary or Pulmonary Neoplasm or Cancer*, Lung or Pulmonary Cancer* or Cancer*, Pulmonary or Cancer of the Lung or Cancer of Lung or Small Cell Lung Cancer or Oat Cell Lung Cancer or Small Cell Cancer Of The Lung or Carcinoma, Small Cell Lung or Oat Cell Carcinoma of Lung or Lung Adenocarcinoma* or Adenocarcinoma*, Lung or Adenocarcinoma*, Bronchiolo Alveolar or Bronchiolo-Alveolar Adenocarcinoma* or Adenocarcinoma*, Alveolar or Alveolar Adenocarcinoma* or Carcinoma*, Alveolar or Alveolar Carcinoma* or Bronchiolo-Alveolar Carcinoma* or Alveolar Cell Carcinoma* or Carcinoma*, Alveolar Cell or Bronchiolar Carcinoma* or Carcinoma*, Bronchiol* or Bronch* Carcinoma* or Neoplasm*, Bronchial or Bronchial Neoplasm or Carcinoma*, Bronch* or Bronchial Carcinoma* or Multiple Pulmonary Nodule or Pulmonary Nodule*, Multiple or Syndrome, Pancoast* or Pancoast* Syndrome or Pancoast Tumor or Tumor, Pancoast or Blastoma*, Pulmonary or Pulmonary Blastomas or Pulmonary Sclerosing Hemangiomas or Sclerosing Hemangioma*, Pulmonary or Lung Sclerosing Hemangioma* or Sclerosing Hemangioma*, Lung or Hemangioma, Sclerosing, Pulmonary or Sclerosing Hemangioma of the Lung or Carcinoma, Squamous Cell or Carcinoma, Large Cell or Carcinoma, Small Cell **100025**

**#3** AB Carcinoma*, Non-Small-Cell Lung or Lung Neoplasms or Small Cell Lung Carcinoma or Adenocarcinoma of Lung or Adenocarcinoma, Bronchiolo-Alveolar or Bronchial Neoplasms or Carcinoma, Bronchogenic or Multiple Pulmonary Nodules or Pancoast Syndrome or Pulmonary Blastoma or Pulmonary Sclerosing Hemangioma or lung cancer* or lung tumo* or non-small-cell lung* or NSCLC or SCLC or small-cell lung cancer or bronchogenic carcinoma or Primary bronchogenic carcinoma or Lung carcinoma or Small cell lung cancer or Carcinoma, Non Small Cell Lung or Lung Carcinoma*, Non-Small-Cell or Non-Small-Cell Lung Cancer* or Lung Carcinoma* or Non small Cell Lung Cancer or Non Small Cell Lung Carcinoma or Carcinoma, Non-Small Cell Lung or Pulmonary Neoplasms or Neoplasm*, Lung or Lung Neoplasm or Neoplasm*, Pulmonary or Pulmonary Neoplasm or Cancer*, Lung or Pulmonary Cancer* or Cancer*, Pulmonary or Cancer of the Lung or Cancer of Lung or Small Cell Lung Cancer or Oat Cell Lung Cancer or Small Cell Cancer Of The Lung or Carcinoma, Small Cell Lung or Oat Cell Carcinoma of Lung or Lung Adenocarcinoma* or Adenocarcinoma*, Lung or Adenocarcinoma*, Bronchiolo Alveolar or Bronchiolo-Alveolar Adenocarcinoma* or Adenocarcinoma*, Alveolar or Alveolar Adenocarcinoma* or Carcinoma*, Alveolar or Alveolar Carcinoma* or Bronchiolo-Alveolar Carcinoma* or Alveolar Cell Carcinoma* or Carcinoma*, Alveolar Cell or Bronchiolar Carcinoma* or Carcinoma*, Bronchiol* or Bronch* Carcinoma* or Neoplasm*, Bronchial or Bronchial Neoplasm or Carcinoma*, Bronch* or Bronchial Carcinoma* or Multiple Pulmonary Nodule or Pulmonary Nodule*, Multiple or Syndrome, Pancoast* or Pancoast* Syndrome or Pancoast Tumor or Tumor, Pancoast or Blastoma*, Pulmonary or Pulmonary Blastomas or Pulmonary Sclerosing Hemangiomas or Sclerosing Hemangioma*, Pulmonary or Lung Sclerosing Hemangioma* or Sclerosing Hemangioma*, Lung or Hemangioma, Sclerosing, Pulmonary or Sclerosing Hemangioma of the Lung or Carcinoma, Squamous Cell or Carcinoma, Large Cell or Carcinoma, Small Cell **100025**

**#4 #1 OR #2 OR #3 100025**

**#5** MM Mass Screening OR MM Early Detection of Cancer OR MM Early Diagnosis OR MM Multiphasic Screening OR MM Mass Chest X-Ray OR MM Tomography, Spiral Computed **10178**

**#6** TI Screen or Diagnosis or Detection or Test or Tomography or ray **1995763**

**#7** AB Screen or Diagnosis or Detection or Test or Tomography or ray **2003884**

**#8 #5 OR #6 OR #7 2003884**

**#9** MM Tobacco Use Disorder **0**

**#10** TI Tobacco or Cigarette or Smok* or Nicotine **139145**

**#11** AB Tobacco or Cigarette or Smok* or Nicotine **143199**

**#12 #9 OR #10 OR #11 143199**

**#13** MM ( Acupuncture or Anti-Anxiety Agents or Antidepressive Agents or Antidepressive Agents, Second-Generation or Azocines or Behavior Therapy or Benzazepines or Bupropion or Counseling or Directive Counseling or Exercise or Hypnosis ) OR MM ( Nicotinic Agonists or Nicotinic Antagonists or Nortriptyline or Patient Education as Topic or Quinolizines or Quinoxalines or Electronic Nicotine Delivery Systems or Vaping ) **76214**

**#14** TI Acupuncture or Anti-Anxiety Agents or Antidepressive Agents or Antidepressive Agents, Second-Generation or Azocines or Behavior Therapy or Benzazepines or Bupropion or Counseling or Directive Counseling or Exercise or Hypnosis or Nicotinic Agonists or Nicotinic Antagonists or Nortriptyline or Patient Education as Topic or Quinolizines or Quinoxalines or Electronic Nicotine Delivery Systems or Vaping or abstinen* or antidepressant* or fluoxetin* or imipramin* or anxiolyti* or Bupropion or cessation aid* or clonidine or cytisine or Dianicline or doxepin* or lazabemide* or lobeline or mecamylamine or moclobemide or nicobrevin* or nicotine gum* or nicotine inhaler* or nicotine nasal spray* or nicotine patch* or nicotine therap* or nicotine vaccin* or NRT or nicotine replacement therap* or paroxetin* or patient education or quit smoking or quitting smok* or rimonabant or selegilin* or sertralin* or silver acetat* or stop smoking or stopping smoking **511732**

**#15** AB Acupuncture or Anti-Anxiety Agents or Antidepressive Agents or Antidepressive Agents, Second-Generation or Azocines or Behavior Therapy or Benzazepines or Bupropion or Counseling or Directive Counseling or Exercise or Hypnosis or Nicotinic Agonists or Nicotinic Antagonists or Nortriptyline or Patient Education as Topic or Quinolizines or Quinoxalines or Electronic Nicotine Delivery Systems or Vaping or abstinen* or antidepressant* or fluoxetin* or imipramin* or anxiolyti* or Bupropion or cessation aid* or clonidine or cytisine or Dianicline or doxepin* or lazabemide* or lobeline or mecamylamine or moclobemide or nicobrevin* or nicotine gum* or nicotine inhaler* or nicotine nasal spray* or nicotine patch* or nicotine therap* or nicotine vaccin* or NRT or nicotine replacement therap* or paroxetin* or patient education or quit smoking or quitting smok* or rimonabant or selegilin* or sertralin* or silver acetat* or stop smoking or stopping smoking **509864**

**#16 #13 OR #14 OR #15 509864**

**#17 #4 AND #8 AND #12 AND #16 761**

**#18** MM Quantitative Studies+ OR Clinical Trials+ OR Randomized Controlled Trials OR Random Assignment OR Placebos **266410**

**#19** AB random* OR crossover* OR (cross N3 over*) OR placebo OR (doubl* N3 blind*) OR (doubl* N3 mask*) OR (singl* N3 blind*) OR (singl* N3 mask*) OR (trebl* N3 blind*) OR (trebl* N3 mask*) OR (tripi* N3blind*) OR (tripl* N3 mask*) OR assign* OR allocat* OR volunteer* **548822**

**#20** TI random* OR crossover* OR (cross N3 over*) OR placebo OR (doubl* N3 blind*) OR (doubl* N3 mask*) OR (singl* N3 blind*) OR (singl* N3 mask*) OR (trebl* N3 blind*) OR (trebl* N3 mask*) OR (tripi* N3blind*) OR (tripl* N3 mask*) OR assign* OR allocat* OR volunteer* **395241**

**#21 #18 OR #19 OR #20 395241**

**#22 #17 AND #21 53**

## 1.3.6 PsycINFO 2022-02-07 (35 items)

**#1** (Carcinoma, Non-Small-Cell Lung or Small Cell Lung Carcinoma or Lung Neoplasms or Adenocarcinoma of Lung or Adenocarcinoma, Bronchiolo-Alveolar or Bronchial Neoplasms or Carcinoma, Bronchogenic or Multiple Pulmonary Nodules or Pancoast Syndrome or Pulmonary Blastoma or Pulmonary Sclerosing Hemangioma).mh. **1346**

**#2** (Carcinoma*, Non-Small-Cell Lung or Lung Neoplasms or Small Cell Lung Carcinoma or Adenocarcinoma of Lung or Adenocarcinoma, Bronchiolo-Alveolar or Bronchial Neoplasms or Carcinoma, Bronchogenic or Multiple Pulmonary Nodules or Pancoast Syndrome or Pulmonary Blastoma or Pulmonary Sclerosing Hemangioma or lung cancer* or lung tumo* or non-small-cell lung* or NSCLC or SCLC or small-cell lung cancer or bronchogenic carcinoma or Primary bronchogenic carcinoma or Lung carcinoma or Small cell lung cancer or Carcinoma, Non Small Cell Lung or Lung Carcinoma*, Non-Small-Cell or Non-Small-Cell Lung Cancer* or Lung Carcinoma* or Non small Cell Lung Cancer or Non Small Cell Lung Carcinoma or Carcinoma, Non-Small Cell Lung or Pulmonary Neoplasms or Neoplasm*, Lung or Lung Neoplasm or Neoplasm*, Pulmonary or Pulmonary Neoplasm or Cancer*, Lung or Pulmonary Cancer* or Cancer*, Pulmonary or Cancer of the Lung or Cancer of Lung or Small Cell Lung Cancer or Oat Cell Lung Cancer or Small Cell Cancer Of The Lung or Carcinoma, Small Cell Lung or Oat Cell Carcinoma of Lung or Lung Adenocarcinoma* or Adenocarcinoma*, Lung or Adenocarcinoma*, Bronchiolo Alveolar or Bronchiolo-Alveolar Adenocarcinoma* or Adenocarcinoma*, Alveolar or Alveolar Adenocarcinoma* or Carcinoma*, Alveolar or Alveolar Carcinoma* or Bronchiolo-Alveolar Carcinoma* or Alveolar Cell Carcinoma* or Carcinoma*, Alveolar Cell or Bronchiolar Carcinoma* or Carcinoma*, Bronchiol* or Bronch* Carcinoma* or Neoplasm*, Bronchial or Bronchial Neoplasm or Carcinoma*, Bronch* or Bronchial Carcinoma* or Multiple Pulmonary Nodule or Pulmonary Nodule*, Multiple or Syndrome, Pancoast* or Pancoast* Syndrome or Pancoast Tumor or Tumor, Pancoast or Blastoma*, Pulmonary or Pulmonary Blastomas or Pulmonary Sclerosing Hemangiomas or Sclerosing Hemangioma*, Pulmonary or Lung Sclerosing Hemangioma* or Sclerosing Hemangioma*, Lung or Hemangioma, Sclerosing, Pulmonary or Sclerosing Hemangioma of the Lung or Carcinoma, Squamous Cell or Carcinoma, Large Cell or Carcinoma, Small Cell).ti. **1222**

**#3** (Carcinoma*, Non-Small-Cell Lung or Lung Neoplasms or Small Cell Lung Carcinoma or Adenocarcinoma of Lung or Adenocarcinoma, Bronchiolo-Alveolar or Bronchial Neoplasms or Carcinoma, Bronchogenic or Multiple Pulmonary Nodules or Pancoast Syndrome or Pulmonary Blastoma or Pulmonary Sclerosing Hemangioma or lung cancer* or lung tumo* or non-small-cell lung* or NSCLC or SCLC or small-cell lung cancer or bronchogenic carcinoma or Primary bronchogenic carcinoma or Lung carcinoma or Small cell lung cancer or Carcinoma, Non Small Cell Lung or Lung Carcinoma*, Non-Small-Cell or Non-Small-Cell Lung Cancer* or Lung Carcinoma* or Non small Cell Lung Cancer or Non Small Cell Lung Carcinoma or Carcinoma, Non-Small Cell Lung or Pulmonary Neoplasms or Neoplasm*, Lung or Lung Neoplasm or Neoplasm*, Pulmonary or Pulmonary Neoplasm or Cancer*, Lung or Pulmonary Cancer* or Cancer*, Pulmonary or Cancer of the Lung or Cancer of Lung or Small Cell Lung Cancer or Oat Cell Lung Cancer or Small Cell Cancer Of The Lung or Carcinoma, Small Cell Lung or Oat Cell Carcinoma of Lung or Lung Adenocarcinoma* or Adenocarcinoma*, Lung or Adenocarcinoma*, Bronchiolo Alveolar or Bronchiolo-Alveolar Adenocarcinoma* or Adenocarcinoma*, Alveolar or Alveolar Adenocarcinoma* or Carcinoma*, Alveolar or Alveolar Carcinoma* or Bronchiolo-Alveolar Carcinoma* or Alveolar Cell Carcinoma* or Carcinoma*, Alveolar Cell or Bronchiolar Carcinoma* or Carcinoma*, Bronchiol* or Bronch* Carcinoma* or Neoplasm*, Bronchial or Bronchial Neoplasm or Carcinoma*, Bronch* or Bronchial Carcinoma* or Multiple Pulmonary Nodule or Pulmonary Nodule*, Multiple or Syndrome, Pancoast* or Pancoast* Syndrome or Pancoast Tumor or Tumor, Pancoast or Blastoma*, Pulmonary or Pulmonary Blastomas or Pulmonary Sclerosing Hemangiomas or Sclerosing Hemangioma*, Pulmonary or Lung Sclerosing Hemangioma* or Sclerosing Hemangioma*, Lung or Hemangioma, Sclerosing, Pulmonary or Sclerosing Hemangioma of the Lung or Carcinoma, Squamous Cell or Carcinoma, Large Cell or Carcinoma, Small Cell).ab. **2988**

**#4 #1 OR #2 OR #3 3333**

**#5** (Mass Screening or Early Detection of Cancer or Early Diagnosis or Multiphasic Screening or Mass Chest X-Ray or Tomography, Spiral Computed).mh. **13657**

**#6** (Screen* or Diagnosis or Detection or Test* or Compute* Tomography or CT or ray).ti. **141958**

**#7** (Screen* or Diagnosis or Detection or Test* or Compute* Tomography or CT or ray).ab. **958762**

**#8 #5 OR #6 OR #7 985759**

#**9** Tobacco Use Disorder.mh. **0**

**#10** (Tobacco or Cigarette or Smok* or Nicotine).ti. **37483**

**#11** (Tobacco or Cigarette or Smok* or Nicotine).ab. **71274**

**#12 #9 OR #10 OR #11 72022**

**#13** (Acupuncture or Anti-Anxiety Agents or Antidepressive Agents or Antidepressive Agents, Second-Generation or Azocines or Behavior Therapy or Benzazepines or Bupropion or Counseling or Directive Counseling or Exercise or Hypnosis).mh. **54695**

**#14** (Nicotinic Agonists or Nicotinic Antagonists or Nortriptyline or Patient Education as Topic or Quinolizines or Quinoxalines or Electronic Nicotine Delivery Systems or Vaping).mh. **15793**

**#15 #13 OR #14 68188**

**#16** (Acupuncture or Anti-Anxiety Agents or Antidepressive Agents or Antidepressive Agents, Second-Generation or Azocines or Behavior Therapy or Benzazepines or Bupropion or Counseling or Directive Counseling or Exercise or Hypnosis or Nicotinic Agonists or Nicotinic Antagonists or Nortriptyline or Patient Education as Topic or Quinolizines or Quinoxalines or Electronic Nicotine Delivery Systems or Vaping or abstinen* or antidepressant* or fluoxetin* or imipramin* or anxiolyti* or Bupropion or cessation aid* or clonidine or cytisine or Dianicline or doxepin* or lazabemide* or lobeline or mecamylamine or moclobemide or nicobrevin* or nicotine gum* or nicotine inhaler* or nicotine nasal spray* or nicotine patch* or nicotine therap* or nicotine vaccin* or NRT or nicotine replacement therap* or paroxetin* or patient education or quit smoking or quitting smok* or rimonabant or selegilin* or sertralin* or silver acetat* or stop smoking or stopping smoking).ti. **67665**

**#17** (Acupuncture or Anti-Anxiety Agents or Antidepressive Agents or Antidepressive Agents, Second-Generation or Azocines or Behavior Therapy or Benzazepines or Bupropion or Counseling or Directive Counseling or Exercise or Hypnosis or Nicotinic Agonists or Nicotinic Antagonists or Nortriptyline or Patient Education as Topic or Quinolizines or Quinoxalines or Electronic Nicotine Delivery Systems or Vaping or abstinen* or antidepressant* or fluoxetin* or imipramin* or anxiolyti* or Bupropion or cessation aid* or clonidine or cytisine or Dianicline or doxepin* or lazabemide* or lobeline or mecamylamine or moclobemide or nicobrevin* or nicotine gum* or nicotine inhaler* or nicotine nasal spray* or nicotine patch* or nicotine therap* or nicotine vaccin* or NRT or nicotine replacement therap* or paroxetin* or patient education or quit smoking or quitting smok* or rimonabant or selegilin* or sertralin* or silver acetat* or stop smoking or stopping smoking).ab. **201106**

**#18 #15 OR #16 OR #17 244250**

**#19 #4 AND #8 AND #12 AND #18 35**

**#20** (randomized controlled trial or controlled clinical trial or randomized or randomised or placebo or sham or randomly or trial or groups).ti. **64607**

**#21** (randomized controlled trial or controlled clinical trial or randomized or randomised or placebo or sham or randomly or trial or groups).ab. **641433**

**#22 #20 OR #21 649743**

**#23 19 AND #22 35**

## 1.3.7 Science Direct 2022-02-07(19 items)

**#1** Title, abstract, keywords: “lung cancer” OR “lung tumour” OR “Pulmonary Cancer” OR “Neoplasm, Lung” **78702**

**#2** Title, abstract, keywords:“Early diagnosis” OR “Primary diagnosis” OR “Screening” **227373**

**#3** Title, abstract, keywords: “Tobacco Use Disorder” OR “Cigarette smoker” **1978**

**#4 #1 AND #2 AND #3 19**

# Registration and Protocol

We made some amendments to the original protocol. The systematic review took so long a time to complete that our complete date surpass the anticipated one. The Prospero platform did not allow us to make any changes. So we present the changes we made here.

**Table 1. Amendments to the original protocol.**

| Domain | Original information in PROSPERO | After changes |
| --- | --- | --- |
| 1. Anticipated completion date | 2019.12 | 2022.7 |
| 2. Review team members and their organizational affiliations | Ms Xinxin Ye. Ms Simin Huang. Ms Oufeng Tang. Mr Zhong Bo Xu. Ms Qixi Liu. Professor Chenju Zhan. Mr Yuzhen Gao. | Simin Huang, MD; Oufeng Tang, MD; Xutong Zheng, MD; Hui Li, MD; Yuxin Wu, MD; Li Ge, PhD; Liu Yang, MD |
| 3. Searches |  |  |
| （1）time limit of searching | 2019.6 | 2022.1 |
| （2）Inclusion criteria: | ①Studies including smokers in lung screening.  ②Studies in which any counseling, digital, pharmacological, provider, or health system-based smoking  cessation intervention.  ③Studies in which the estimated results of the patients have been reported as 7-day point prevalence of  smoking cessation Biochemically confirmed smoking abstinence rates, Quit attempts rates, point-prevalence  abstinence, 30-day abstinence rates (at one-month, three-month, twelve-month and two-year), and number  of tobacco cigarettes smoked. | ①design in RCTs;  ②participants: smokers who smoked ≥30 packs/year and stopped smoking <15 years, regardless of sex;  ③interventions: studies comparing smoking cessation interventions to standard care, including authoritative smoking cessation materials, placebo, or other therapies with similar co-interventions between intervention and control groups; types of smoking cessation interventions can be any network resource, individualized intervention, and replacement therapy;  ④outcome measures: the main outcome measures were smoking abstinence, which can be verified by patient-reported outcome or biochemical verification |
| （3）Exclusion criteria: | ①Studies involving fewer than fifty patients.  ②Studies in non-randomized controlled trials. | ①case reports, case-control studies, reviews, protocols, or animal experimental studies; ②studies without smoking abstinence as the outcome measure;  ③duplicated publications;  ④incomplete or unavailable data. |
| 4. Main outcome(s) | 7-day point prevalence of smoking cessation Biochemically confirmed smoking abstinence rates, Quit  attempts rates, point-prevalence abstinence, 30-day abstinence rates, number of tobacco cigarettes smoked | 7-day-point prevalence of smoking abstinence（which can be verified by patient-reported outcome or biochemical verification）； continuous smoking abstinence（which can be verified by patient-reported outcome or biochemical verification） |
